# The Chicago School Readiness Project: Examining the long-term impacts of an early childhood intervention

**DOI:** 10.1371/journal.pone.0200144

**Published:** 2018-07-12

**Authors:** Tyler W. Watts, Jill Gandhi, Deanna A. Ibrahim, Michael D. Masucci, C. Cybele Raver

**Affiliations:** 1 Steinhardt School of Culture, Education and Human Development, New York University, New York, New York, United States of America; 2 New York State Psychiatric Institute, Division of Translational Imaging, Columbia University Medical Center, New York, New York, United States of America; TNO, NETHERLANDS

## Abstract

The current paper reports long-term treatment impact estimates for a randomized evaluation of an early childhood intervention designed to promote children's developmental outcomes and improve the quality of Head Start centers serving high-violence and high-crime areas in inner-city Chicago. Initial evaluations of end-of-preschool data reported that the program led to reductions in child behavioral problems and gains in measures of executive function and academic achievement. For this report, we analyzed adolescent follow-up data taken 10 to 11 years after program completion. We found evidence that the program had positive long-term effects on students’ executive function and grades, though effects were somewhat imprecise and dependent on the inclusion of baseline covariates. Results also indicated that treated children had heightened sensitivity to emotional stimuli, and we found no evidence of long-run effects on measures of behavioral problems. These findings raise the possibility that developing programs that improve on the Head Start model could carry long-run benefits for affected children.

## Introduction

Early childhood interventions have received substantial policy attention with the hope that providing high quality early educational environments to children from underserved communities can offset the effects of poverty on long-run development [1,2]. Yet, evidence from experimental studies regarding this hypothesis is surprisingly scarce and mixed. Unfortunately, too few studies of early interventions have followed children beyond the elementary school years, leaving questions as to whether early intervention effects should be expected to persist into adolescence or adulthood.

The current study evaluated the long-term effects of the Chicago School Readiness Project (CSRP), a cluster-randomized design preschool intervention that aimed to improve the chances of early school success for children living in high-poverty and high-crime neighborhoods in inner city Chicago. The intervention, which provided teachers with professional development and coaching that targeted student behavioral management and teacher stress reduction, was administered in Head Start classrooms, and it was designed to improve the quality of Head Start while also promoting children’s self-regulation and executive functioning [3]. Initial evaluations showed that the intervention positively affected the quality of the preschool classroom environment [3], as well as measures of children’s self-regulation [4], executive function and academic achievement [5], and it was predicted that these early impacts on child-level skills and behavior would persist in the long-term. In the current paper, we report program impacts on a broad array of adolescent outcomes collected 10 to 11 years after program completion, including measures of executive function, academic achievement, behavioral problems, and emotional regulation.

### Early intervention and long-term effects

An alarming 13.3 million children live below the poverty line in the United States [6], and research suggests that a substantial fraction of these children will face higher rates of emotional, behavioral and mental health problems throughout their lives, including depression and anxiety and greater levels of health and behavioral risk-taking [7–10]. Likewise, children growing up in economically under-resourced, ethnic minority neighborhoods face major disparities in access to higher quality education, with high school graduation rates for African American and Latino students severely lagging behind the national average [11,12]. More troubling, research clearly indicates that spending the early childhood years [typically defined as ages 0 through 5] in poverty substantially increases the risk of detrimental effects on long-term development. Longitudinal studies suggest that exposure to poverty during early childhood, even when accounting for exposure during middle childhood and adolescence, strongly predicts a host of negative adult outcomes, including lower earnings and poor labor market success [13], fewer years of completed schooling [14], and higher rates of obesity [15].

Policymakers and researchers have increasingly turned to early intervention as a means of offsetting the adverse effects of poverty exposure on development (for reviews see [1,2,16,17]). The rationale for such investments is straightforward: If interventions can positively affect children’s cognitive and socioemotional development during the formative years of early childhood, then such effects may place children on more positive lifelong trajectories. However, the evidence in support of this hypothesis is mixed. On the one hand, a small set experimental studies, bolstered by a larger set of quasi-experimental studies, have found positive effects for early childhood interventions on measures of long-term attainment, such as high school graduation rate [18,19]. Conversely, some recent experimental work has found that early childhood interventions often produce substantial positive effects at the time of program completion, only to see these effects fade to 0 in the few years after the intervention ends [20].

This pattern of “fadeout” is best illustrated by the findings reported in a recent meta-analysis by Bailey and colleagues of 67 high-quality early childhood interventions evaluated through randomized control trials published between 1960 and 2007, as the meta-analytic average treatment effect across studies was observed to have a precipitous decline during the 3 years following intervention [20]. This disconcerting pattern of effects suggests that theories that predict that early intervention will lead to sustained changes in children’s long-term trajectories may be flawed or incomplete, and interventions targeted at the earliest stages of development may not be as cost-effective as initially hoped if effects inevitably fade out.

However, there remain compelling reasons to continue to investigate whether successful early interventions may produce effects measured into adolescence or adulthood. First, other reviews and meta-analyses that included quasi-experimental evaluations of early childhood programs have found evidence of positive long-run effects [18,19,21]. For example, Camili and colleagues’ analysis of 123 early childhood interventions, all of which included some type of comparison group, found evidence that early intervention had effects on long-run measures of cognitive ability [18], and a recent meta-analysis by McCoy and colleagues, which also included quasi-experimental work, found that early intervention affected long-term educational outcomes such as high school graduation [19]. Further, three well-documented intervention studies, the Perry Preschool Program [22–25], the Abecedarian Early Intervention Project [26], and the Chicago Child Parent Centers program [27,28], all produced positive effects measured through adolescence and, in some cases, adulthood. Finally, these findings are further supported by correlational studies that have consistently demonstrated the strong relation between early gains in cognitive and socio-emotional skills and long-term developmental outcomes [21,29,30].

Thus, the question of whether long-run effects should be expected following a successful early intervention remains open. Multiple reviews of the literature [18,20,21] have argued that the presence of long-term impacts is likely governed by key program features, such as the types of skills targeted by the intervention and the difference in quality between the environment offered by the program and the environment encountered in the counterfactual. However, these reviews have also noted the need for more experimental intervention evaluations with longitudinal follow-up, as we need additional evidence from interventions with different programmatic features to better understand when long-term effects are likely to be found.

The current study aims to provide new longitudinal experimental evidence to further contribute to the literature base on early childhood interventions, as we analyzed data collected 10 to 11 years after the completion of a successful early childhood intervention that substantially boosted low-income children’s cognitive and socioemotional functioning measured at the end of preschool [4,5].

### The Chicago School Readiness Project

The Chicago School Readiness Project (CSRP) was conceived as an early childhood intervention designed to bolster children’s self-regulation and executive function skills through changing the classroom quality of Head Start centers operating in high-poverty and high-crime neighborhoods within inner-city Chicago. The CSRP intervention model was supported by research that suggested that children’s early educational experiences could be substantially improved by targeting teacher’s behavioral management strategies, and improvements to teacher behavioral management should help improve children’s own self-regulation abilities [31–34]. By changing the way teachers supported the development of children’s early self-regulation, the intervention was expected to help children become more positively engaged, attentive learners during their earliest school experiences, and gains in self-regulation were hypothesized to set children on a higher achieving trajectory throughout school.

The CSRP targeted Head Start centers because of Head Start’s unique role as the primary early education service provider for low-income families in the U.S., and other studies have shown that well-designed programs could be integrated into the Head Start model (e.g., [35–39]). The overarching goal of improving classroom practices and child self-regulation was enacted through a multi-component model, as the CSRP targeted services to the children, families and teachers of participating Head Start centers.

The CSRP targeted children’s self-regulation skills by providing teachers with extensive professional development designed to help them improve their classroom behavioral management. Self-regulation describes a child’s ability to focus and maintain attention, regulate behavior in order to positively interact with peers and adults, and regulate emotion in the face of stress and anxiety [38]. Executive function (EF) is considered to be the cognitive component of self-regulation, and EF involves activation of the sub-areas of the prefrontal cortex. When faced with stress and adversity, children with higher-level executive functioning are better able to plan ahead, maintain focus, and rely on cognitive flexibility to solve difficult problems [39–41]. Not surprisingly, these skills are essential for early school success [34,42–44], as self-regulation supports the acquisition of new information by allowing children to focus and sustain their attention as well as to suppress impulsive responses in favor of better academic engagement [45]. Longitudinal work has also shown that self-regulation skills help students transition into college [46], and children who self-regulate have lower rates of criminal behavior and better health outcomes as adults [29,47].

Because children with exposure to poverty and poverty-related stressors are at greater risk for more self-regulatory difficulty [48,49], CSRP specifically targeted self-regulation as a way to provide children with a skill that could substantially alter their long-term school experiences. Previous research suggests that self-regulation skills are key in shaping children’s early school relationships with peers and teachers [50,51], and children’s early behavioral problems have been implicated as a likely cause of low childcare quality in Head Start centers [52]. Preschool teachers working in low-resource areas are often ill-equipped to deal with the trauma-related behavioral challenges facing children living in impoverished neighborhoods [53], and this lack of training can lead to punitive and coercive behavioral management strategies that may actually exacerbate children’s early behavioral problems [54]. Observational work has also shown that teachers in high-poverty classrooms spend a disproportionate amount of time on behavioral management, taking away crucial time for academic instruction, which may be particularly important for supporting the academic achievement of low-income populations [55]. Thus, by providing teachers with better behavioral management strategies, CSRP was designed to bolster students’ self-regulation skills, while also improving the quality of the classroom environment by supporting the relationships between teachers and children.

Finally, the program also directed services directly at Head Start teachers, with the understanding that teaching in Head Start centers serving communities in high-poverty and high-crime areas could lead to substantial stress and burnout [53,56]. The lack of training and support given to teachers working in severely under-resourced communities has been shown to lead to high rates of teacher turnover, and the low-pay afforded to teachers also makes them less likely to access mental health services for their own psychological wellbeing [57,58]. As such, CSRP provided teachers with access to mental health consultants (MHC), who conducted several stress-reduction workshops throughout the year. The MHC’s each held a master’s degree in social work, and they visited the classroom weekly for a period spanning 4 months to help teachers implement the behavioral management strategies introduced during the student-focused professional development sessions. MHC’s also provided direct intervention services to 3 to 4 children per class. These children had been flagged as having especially severe behavioral or emotional problems, and they were given the opportunity to meet with MHC’s for either individual or group therapy sessions.

Thus, the CSRP program offered a fairly comprehensive approach to improving the quality of the Head Start environment as it targeted both student behavioral and emotional regulation as well as teacher psychological stress and burnout. Initial evaluations suggested that the program was implemented with a high degree of fidelity, and analyses of classroom observational measures indicated that CSRP teachers provided Head Start children with significantly better-managed and more emotionally supportive classrooms when compared with teachers in the control condition [3]. Further, the program affected a broad set of child-level competencies measured at the end of preschool, with evaluations reporting that the program reduced children’s behavioral problems (*d*s = 0.53–0.89) [4] and boosted children’s EF, reading, language, and math skills (*d*s = 0.20–0.63) [5].

This initial evaluation work suggested that through changing the quality of the classroom, and through improving children’s interactions with their teachers, the CSRP boosted measures of self-regulation and also positively affected children’s academic school readiness skills. It was hypothesized that these initial benefits would produce long-lasting impacts on children’s cognitive and socio-emotional trajectories, but follow-up work was mixed and inconclusive. Evidence of fadeout on child measures of behavioral regulation and academic achievement was found in the early years of elementary school [59], though there was some indication that children who attended higher-quality elementary schools may have had longer-lasting intervention benefits. Similarly, a recent follow-up study employed a mixed growth curve modeling approach, and found that the treatment may have caused reductions in the likelihood of following an increasing trajectory of internalizing behavioral problems during elementary school [60], but these effects were relatively small and for only a subset of children. Unfortunately, these prior follow-up studies lacked the broad set of measures used at the end of preschool, making this study the first complete attempt to understand the long-term impacts of the CSRP intervention on the full set of developmental domains originally hypothesized to be affected.

### Current study

In the current study, we analyzed newly-collected measures of adolescent functioning, collected 10 to 11 years after children first participated in the CSRP intervention. These data allowed us to test whether the CSRP program had long-term effects on indicators of children’s executive function, academic achievement, behavioral problems and emotional regulation. We selected this set of measures because it closely reflected the set of measures first used to assess program efficacy at the end of preschool [4,5], and because these measures have been shown to be important indicators of adolescent wellbeing [61,62] and critical predictors of adult attainment [29].

Based on theoretical and empirical work that has shown that the early acquisition of self-regulation and academic achievement skills support children’s long-term cognitive and socio-emotional development [34,29], we expected that the CSRP would have positive impacts on measures of adolescent executive function and achievement, and we also expected that adolescents assigned to the treatment group would have less behavioral problems and a greater capacity for regulating emotion. However, our expectations were tempered by work showing fadeout in the years following early intervention [20], and given the paucity of early interventions with long-term follow-up, we had no strong prediction for the effect sizes that we might detect.

## Method

### Ethics statement

All research procedures and protocols including participant recruitment materials were reviewed and approved by the University Committee on Activities Involving Human Subjects at New York University. Parents of participating subjects provided consent and all participating children and adolescents provided verbal assent.

### Study design

The CSRP program was evaluated through a cluster-randomized design, and Head Start sites were recruited based on four criteria: 1) receipt of federal Head Start funding; 2) having two or more classrooms that offered full-day classes; 3) location in a set of high-poverty Chicago neighborhoods that contained high crime rates, low rates of mobility, and a substantial portion of families living below the poverty line; 4) completion of a screening self-nomination procedure [3]. The recruitment process led to 18 Head Start centers participating in the study, with centers grouped into pairs based on a set of 14 site-level characteristics. Within these pairs, which we subsequently refer to as “blocking groups,” sites were randomly assigned to either treatment or control, and treatment sites implemented the CSRP intervention program described above. Control sites continued “business-as-usual,” but classrooms in control sites were provided with part-time teaching aides to account for the changes in student-to-teacher ratio brought on by MHC’s in treatment sites. The program was implemented for two different cohorts of students and teachers, with Cohort 1 participating in 2004–2005 (57% of the sample) and Cohort 2 participating in 2005–2006.

Two classrooms from each of the 18 Head Start sites were selected for study participation, and evaluators successfully recruited 83% of students from these classes to participate in data collection (student *n* = 602). During the school year, one classroom in the control condition lost Head Start funding due to budget cuts, which resulted in 35 total classrooms participating in the study. At the beginning of the preschool year (i.e., baseline), information regarding the child’s family and home environment was obtained via parent survey, and children’s cognitive, behavioral and emotional functioning were measured via direct assessment. Observers blind to treatment status also rated the quality of the classroom environment at baseline, and teachers responded to surveys that measured their professional and educational experiences, as well as their perceptions of their classroom and school environment. Teachers were also surveyed regarding the behavioral problems of each child participating in the study. In the spring, teachers again evaluated children’s behavioral problems, and study examiners again assessed children’s mathematics, literacy, attention and executive functioning via direct assessment (i.e., post-treatment outcomes).

The participants of the study have been followed into adolescence, and the current study reports on data collected during the 2015–2016 school year, which occurred 11 years after the treatment year for Cohort 1 and 10 years after the treatment year for Cohort 2. By the 2015–2016 follow-up year, 466 adolescents remained in the study (236 in the treatment group and 230 in the control group), and this 23% rate of attrition did not statistically significantly differ between the groups (*p* = 0.51). At the time of the assessment, approximately 70% of participants were in high school, and 30% of the sample remained in middle school. This grade-level difference was largely due to the 2 cohort design of the study, though grade repetition and the substantial variation in student age at baseline (*M* = 4.1 years, *SD* = .65, range: 2.15–6.08) also contributed.

### Intervention

Recall that the CSRP program consisted of 4 key components: 1) professional development to improve teacher behavior management strategies in support of children's self-regulation; 2) MHC classroom visits to assist teachers in implementing the behavioral management program; 3) MHC’s provision of stress reduction workshops; 4) MHC services targeted at children identified as having especially severe emotional and behavioral issues. The intervention lasted 30 weeks, with MHC support occurring throughout the intervention. Full intervention details have been described in previous reports [3–5]. Here, we provide a brief overview of key program features.

#### Professional development

Teachers in the treatment group were provided with 5 professional development sessions staggered throughout the fall and winter months of school year, each lasting approximately 6 hours. These sessions were based on the Incredible Years Teacher Training Program [63], and teachers were given new strategies to help reduce children’s challenging behavioral problems and to support positive, self-regulated behavior. For example, teachers were provided with video exemplars of being on the lookout for the opportunity to reward and praise prosocial behaviors among children whom they viewed as behaviorally difficult or misbehaving. This strategy of “catching your student being good” was demonstrated to staff as a way to break a coercive cycle of dysregulation and negative teacher attention using concrete examples, simple steps and discussion. Sessions were led by licensed clinical social workers, and MHC’s also attended each session to help foster better relationships between MHC’s and study teachers.

#### Mental health consultants

The MHC’s were master’s level social workers who were required to have experience working with high-risk families in early childhood educational settings. MHC’s were recruited such that they had cultural match with teachers and children in the Head Start centers, and most spoke Spanish and English. In the fall and early winter months of the school year (i.e., the first third of the 30-week intervention), the MHC’s first served as coaches and aides to teachers in their efforts to implement the behavioral management program in the classroom, and MHC’s visited treatment classrooms to provide intervention coaching. During mid-winter of the school year (i.e., the second third of the 30-week intervention), MHCs held a stress reduction workshop for teachers at each site, and they also met one-on-one with teachers to discuss job-related stressors and provide strategies for mitigating burnout. Finally, during the last 10 weeks of the intervention, MHC’s worked directly with approximately 3 to 4 children per class who had been identified by teachers and MHC’s as needing individual attention for issues relating to behavioral and emotional dysregulation.

### Follow-up measures

In Table 1, we present each follow-up measure collected next to the analogous outcome measure collected at the end of preschool. This table hilights the conceptual link between the original outcomes assessed and the outcomes considered in the current paper. Unfortunately, we were not able to collect as many measures for each construct as was originally collected at the end of preschool, but as with the end-of-preschool assessments, our follow-up indicators were also measured through both direct assessment and survey. We describe each follow-up measure collected during adolescence in detail below, and we provide information regarding the original end-of-preschool measures in the supplementary materials (S1 Appendix).

**Table 1:** List of measures used in end-of-preschool evaluations and the current paper.

Construct	End-of-Preschool Measure	Adolescent Measure
*Executive Function*	PSRA—Balance Beam	Hearts and Flowers
	PSRA—Pencil Tap	
*Emotional Regulation*	PSRA—Assessor Report	Emotional Go No Go
*Academic Achievement*	Peabody Picture Vocabulary Test	Self-Reported GPA
	Letter Naming Task	
	Early Math Skills assessment	
*Behavioral Problems*	Behavior Problems Index	Risks & Strengths(a)
	Caregiver-Teacher Report Form	
	Penn Interactive Peer Play Scale	

*Note*. The PSRA stands for the Preschool Self-Regulation Assessment [64], and all end-of-preschool measures are described at length in the original evaluation reports [4,5], and we provide a brief description in the supplementary material (S1 Appendix).

All follow-up measures were completed as part of a 60- to 90-minute computerized assessment battery programmed using Inquisit 4.0.8, a psychological measurement software capable of being tailored to execute various types of assessments [65]. The Inquisit software was programmed to include measures of executive function, emotional regulation, and behavioral problems into the battery. The battery was then presented on HP Stream 11.6-inch notebooks. Programming the battery into laptops allowed participants to be assessed across a range of accessible settings. The battery was administered to participants in the Chicago metropolitan area by trained assessors at school or at home, depending on participants’ and schools’ availability. Out-of-area participants were guided to install and complete the battery on their own computers by trained assessors over the phone or web conference.

#### Executive function

The Hearts and Flowers task (H&F; originally called the “Dots Task”) was used as the primary measure of adolescent executive function [66], as the assessment taps working memory, cognitive flexibility, and inhibitory control [67]. The task asks students to respond to stimuli presented on a screen, and as the task progresses, students are forced to juggle an increasingly difficult set of demands that place stress on their attention and inhibitory control [67]. The task has been used as an overall measure of executive function during adolescence [68], and it has been shown to be a valid measure of executive function as task performance correlates strongly with other measures of working memory and inhibitory control [69].

This measure was the first task in the computerized assessment battery, and children were instructed to respond to the presentation of stimuli on the screen by pressing a key (“Q” or “P”). Stimuli took the form of either hearts or flowers, and they appeared in succession on opposite sides of the screen. When presented with a heart, students were told to press on the same side as the stimulus (“Q” when displayed on the left, “P” when on the right), and when presented with a flower, they were instructed to press on the opposite side (“P” when displayed on the left, “Q” when on the right). Adolescents were given practice trials, and the task began with a series of 12 “hearts only” (congruent) trials, followed by 12 “flowers only” (incongruent) trials. In the final block, adolescents were presented with 33 “mixed trials” including both hearts and flowers stimuli, which substantially increased the difficulty of the task.

In the current study, Hearts and Flowers stimuli were randomly presented on the right or left side for an equal number of trials in each block, and the task took approximately 2 minutes to complete. When interpreting student performance on the task, we focused on mixed block performance, as this has been shown to pose the greatest challenge through the cognitive demand of switching mindsets [69,70]. We used the proportion of correct responses (i.e., the number of trials with a correct response divided by the total number of trials) during mixed block as a measure of working memory, cognitive flexibility and inhibitory control. We also used mean reaction time on mixed trials minus mean reaction time on “hearts only” trials (i.e., the easiest trials) as a measure of the effect of increased cognitive demand on basic processing speed. These two measures are commonly derived from the H&F task, and the H&F task has been used in other early childhood intervention evaluations [66].

#### Academic achievement

We used self-reported GPA as our primary measure of adolescent academic achievement. Students responded to the question “How would you describe your grades in school,” and they were given the following set of options: “mostly A’s,” “mostly B’s,” “mostly C’s,” “mostly D’s,” “mostly F’s,” “none of these grades,” and “not sure.” We coded “none of these grades” and “not sure” responses to missing, and set the remaining options to a 4-point GPA scale (e.g., “mostly A’s” was coded as “4” etc.).

Although we hoped to model outcomes on measures of GPA taken from district offices, administrative data were missing for most students. For the 172 students that had both self-reported GPA and district-reported GPA, these two measures of student grades had a 0.67 correlation. For the 172 students with both forms of data, we found no differences in reporting accuracy between the treatment and control group. We found that a minority of students (*n* = 19) reported “mostly F’s” or “mostly D’s” despite having administrative records showing grades closer to a “C” average. We then recoded these 19 outlier cases to a “C” average, which provided a small improvement to the correlation between self-reported GPA and district-reported GPA (r(172) = 0.68). Thus, our final measure of self-reported GPA was on a 2 to 4 scale, which essentially created a measure with “low,” “middle,” or “high” categories. In the supplementary material (S2 Appendix), we describe our analytic efforts to validate the self-reported measure with the administrative data available, and we describe models that tested whether our main GPA findings were sensitive to our decision to recode the 19 “mostly F’s” and “mostly D’s” cases to “mostly C’s” (results did not substantively differ based on this recoding choice).

#### Behavioral problems

Internalizing and externalizing behaviors were measured through student self-report on the Risks and Strengths Scale, an adapted version of the Children’s Health Risk Behavior Scale [71], which was administered as the third task in the computerized assessment battery. On the internalizing subscale, students responded “yes” or “no” to items asking whether they felt safe, felt bad or scared due to how a peer or adult was treating them, felt unhappy, sad, or depressed, felt worthless or inferior, or felt that they had been crying too much. Similarly, students responded either “yes” or “no” to items on the externalizing subscale, which asked whether or not students had been involved in a physical fight, had gone out with or kissed a boy or girl, had a strong temper, were impulsive, or tried to break or destroy something. Internalizing and externalizing outcome variables were calculated by averaging scores for the items within each subscale. Thus, scores on the measures represent the proportion of times a student indicated that they engaged in either externalizing or internalizing behaviors. Both subscales were reliable measures for our sample, with a Cronbach’s alpha of .74 for internalizing and .67 for externalizing.

#### Emotional regulation

Our measure of adolescent emotional regulation was the Emotional Go/No Go task (EGNG) [72]. Given the emotional changes and instability associated with adolescence [73], it was important to administer a measure that could tap into inhibitory control skills specifically in the face of emotional stimuli. The EGNG task has been validated alongside neuroimaging techniques to display associations between task performance and neurological activity known to play a role in emotional processing [74,75]. The measure is designed to illuminate whether children recognize emotionally expressive faces, and whether the presence of an emotionally expressive face distracts them from focusing on a cognitively challenging task.

In the current study, EGNG was administered as the second task in the computerized assessment battery, and much like the Hearts and Flowers task, it contained trials in which adolescents were presented with stimuli and asked to press a button in response to a stimulus. Stimuli consisted of faces in the center of the screen displaying either happy, sad, angry, or neutral emotions. In each block, neutral faces and faces of one other emotional type were displayed. Before each block, instructions asked participants to respond by pressing the spacebar to either the emotional or neutral faces (“Go” trials), and to withhold responses to the other type of face (“No Go” trials). In addition to a practice block, the task consisted of 6 test blocks—3 Emotional (Happy, Angry or Sad) “Go” versus Neutral “No Go” blocks, and 3 Neutral “Go” versus Emotional “No Go” blocks. Block order was randomized. Each block contained 21 (70%) “Go” response trials and 9 (30%) “No Go” no-response trials. The 70% to 30% Go/No Go trial ratio was implemented to prime participants to respond, making it more difficult for participants to inhibit responding, thus assessing their ability to regulate in response to emotional versus neutral stimuli. Each trial consisted of a 500ms pre-trial pause followed by a 1 second response window, during which the stimulus was presented for 500ms before a 500ms blank screen. The task contained a total of 180 test trials, taking about 6 minutes to complete.

Our analyses focused on measures of performance taken from the four blocks in which participants viewed “Angry vs. Neutral” and “Sad vs. Neutral” faces. The data for this task were organized along three dimensions: hit rate, false alarm rate, and reaction time. Hit rate was the proportion of “Emotion Go” trials answered correctly. For example, “Angry Hit Rate” would be the proportion of trials correctly answered during the “Emotion Go- Angry vs. Neutral” block. False alarm rate was the proportion of “Emotion No Go” trials answered incorrectly (e.g., “Angry False Alarm Rate” would measure the proportion of times a participant responded to angry faces when instructed to respond to neutral faces). Reaction time was a measure of processing speed to emotional stimuli, and it was calculated as the average reaction time on correct hits during “Emotion Go” trials. These three dimensions have been leveraged to understand the role of emotional response inhibition in other low-income samples of children [76].

For “Angry” and “Sad” trials, we then calculated two measures of performance for our treatment impact analyses. The first was D-prime, which has been treated as the primary measure of emotion regulation in previous analyses of EGNG [72], and it was calculated as the standardized difference between emotion-specific hit rate and false alarm rate (e.g., “Angry D-Prime” was calculated as the difference between “Angry Hit Rate” (standardized) and “Angry False Alarm Rate” (standardized)). Finally, as with Hearts and Flowers, we calculated respective measures of adjusted reaction time, which were calculated as reaction time during “Emotion Go” trials for either angry or sad faces minus reaction time during happy “Emotion Go” trials.

### Baseline measures

In the supporting information (S3 Appendix), we present a complete list of all baseline measures included in our treatment impact analyses. These characteristics, all assessed in the fall of the Head Start year, have been described at length in previous reports [4,5]. Here, we present a brief overview of each measure.

#### Child demographic covariates

Child-level demographic characteristics used in the analysis were collected from parents, Head Start site directors, and children themselves, and these characteristics included gender, age at preschool entry, and child ethnicity (White, African American, Hispanic, multiracial, or other).

#### Family/Parent covariates

Upon signing the CSRP consent form for his or her child, the parent or guardian completed a demographic interview. Family and parent characteristics used in the analyses included covariates related to family size, government assistance/support, immigrant status, parent employment, education, marital or partnership status, if the parent was African American or Hispanic, and the biological parent’s contact with the child. Income was represented via an income to needs ratio, calculated as the total family income from the previous year divided by that same year’s federal poverty threshold.

#### Child baseline skills and behavior

Children’s self-regulatory skills and pre-academic skills were collected individually by a group of master’s level assessors who were blind to the treatment status of the children. Measures of executive function and effortful control were collected using the Preschool Self-Regulation Assessment (PSRA) [64], which involved direct assessment of children’s performance levels or latencies on lab-based tasks that were adapted for field administration using paper, pencils, digital timers, and other materials. Executive function was measured with the Balance Beam task [77] and Pencil Tap [78]. Effortful control skills were measured using four delay tasks: Toy Wrap, Toy Wait, Snack Delay, and Tongue Task [77]. Children’s performance across the executive function tasks and the effortful control tasks were standardized and then averaged into two composites. The 28-item PSRA Assessor Report captured global dimensions of children’s impulsivity, attention, and emotions. Two factors representing Attention/Impulse Control and Positive Emotion emerged from the full report, with the Attention/Impulse Control subscale reliably representing children’s self-regulation (internal consistency of α = 0.92).

Children’s vocabulary skills were assessed using the 24-item Peabody Picture Vocabulary Test (PPVT) [79,80] if they spoke English, and the Test de Vocabulario en Imagenes Peabody (TVIP) [81] if they were Spanish-proficient or bilingual. Children’s pre-academic skills were measured via an assessment developed for Head Start that included tests of both letter naming and early math ability [82]. With the letter-naming task, letters of the alphabet were arranged in approximate order of item difficulty, and children were asked to name each letter presented. The early math skills portion of the assessment contained 19 items that covered children’s mastery of counting and basic operations [82].

Children’s behavior problems were rated in the fall by teachers and teaching assistants using the Behavior Problems Index (BPI) [83], a 28-item scale modified for use by teachers. Items were summed into internalizing (α = 0.80) and externalizing (α = 0.92) subscales, and children’s scores were averaged across the child’s teacher and TA. Parents also reported their children’s behavior using the BPI, and ratings of internalizing and externalizing problems from both teachers and parents are included in this analysis.

#### Classroom/Teacher-level covariates

Head Start teacher characteristics were assessed through teacher report and observer ratings in the fall. Teachers reported on their age, level of education, and on several psychosocial characteristics that may influence their perception of their students’ behavioral difficulty. Teachers completed the 6-item K6, a scale of psychological distress [84], the 6-item Job Demands, and the 5-item Job Control subscales of the Child Care and Early Education Job Inventory [85]. These variables were averaged across all teachers in the classroom.

Classroom quality was collected with observational measures in the fall using four subscales of the Classroom Assessment Scoring System (CLASS) [86] and the Early Childhood Environment Rating Scale–Revised (ECERS-R) [87]. The CLASS was used to measure teacher sensitivity, positive climate, negative climate, and behavior management. Finally, class size and number of adults in the class were also added as covariates.

### Analytic approach

We hypothesized that the CSRP intervention would have impacts on our measures of executive function, academic achievement, behavioral problems, and emotional regulation. To test our hypotheses, we began by regressing each dependent variable on treatment status and a series of blocking group fixed effects:

$${Outcome}_{ij}=a_{1}+\beta_{1}{Tx}_{ij}+\sum_{j=1}^{9}\pi \;{Block}_{j}+e_{ij}$$ 1

where *Outcome*_ij_ represents a respective measure of adolescent executive functioning, academic achievement, behavioral problems, or emotional functioning for the *i*th child in blocking group *j* and *Tx*_ij_ represents the treatment status dummy indicator (coded “1” for treatment and “0” for control). We included a series of blocking group fixed effects to account for the cluster-randomized design of the study, and including the series of blocking groups also controls for cohort status, as each block was either in cohort 1 or cohort 2. In this equation, *β*_1_ represents the treatment impact, which will be unbiased only if the error term, *e_ij_*, is uncorrelated with treatment assignment. In other words, our treatment effect estimate would only be unbiased if random assignment produced groups completely balanced on observable and unobservable characteristics.

Because we found evidence of differences between the treatment and control group at baseline (see further description below), we rely on regression models that include covariates for the host of characteristics assessed during the fall of the Head Start year:

$${Outcome}_{ij}=a_{1}+\beta_{1}{Tx}_{ij}+\sum_{j=1}^{9}\pi \;{Block}_{j}+\chi {Child}_{ij}+\lambda {Family}_{ij}+\Omega {Teacher}_{ij}+e_{ij}$$ 2

where *Outcome*_ij_ and *Tx*_ij_ are defined as before, but *Child_ij_*, *Family_ij_*, and *Teacher_ij_* represent sets of controls for child, family, and teacher characteristics all assessed at baseline (see S4 Appendix for complete list). For *Eq 2, β*_1_ will capture the unbiased treatment effect if the baseline control variables account for all observed and unobserved baseline differences between the treatment and control groups.

The estimates generated by *Eq 2*represent our preferred estimates, as these estimates take into account the cluster-design of the study by controlling for blocking group, and they also represent the best attempt to adjust for differences present at baseline by including covariates. With this regression model, we include the full set of baseline covariates in order to generate the most precise estimates possible and to control for any unmeasured source of confounding that could be correlated with measured observables [88,89]. Further, we adjusted standard errors for site-level clustering using the Huber-White adjustment in Stata 15.0, and we used multiple imputation to account for all missing data on baseline covariates. For multiple imputation, we generated 25 multiply imputed datasets using the multivariate normal regression procedure in Stata 15.0 [90].

We also present results from supplementary analyses described below, including estimates that were generated by regression models that adjusted for study attrition, and we provide a host of sensitivity checks in the supplementary information files to ensure that results were not generated due to idiosyncratic features of the statistical models we chose to adopt.

### Data sharing

An anonymized version of the dataset used for the current paper has been made available at datadryad.org (INSERT FINAL WEBSITE URL HERE). The data have been posted along with two additional files: 1) a “readme” explaining the variables contained within the dataset; 2) a file containing the Stata 15.0 syntax that was used to generate the results tables shown in the main text and supplementary material.

## Results

### Baseline equivalence

We began by evaluating baseline equivalence on each measure collected at the beginning of preschool. In Table 2, we present a selection of the pre-treatment measures available to provide a sense of the similarities and differences between the treatment and control groups at baseline, and in supplementary material (S3 Appendix), we provide the complete list of all baseline covariates included in our treatment impact models. Following the recommendations of CONSORT 2010 [see description by de Boer and colleagues, 89], we do not present p-values measuring differences between each individual characteristic. Rather, we focus on describing the general pattern of differences between the groups, and the F-statistic in Table 2 provides an overall measure of the degree to which the groups differed.

**Table 2:** Selected baseline characteristics.

	Treatment	Control

*Demographic*, *Parent and Family Characteristics*		
Female	0.49	0.58
Age (years) on Jan 1 during prek	4.93	4.96
African American	0.67	0.64
Hispanic	0.28	0.27
Bi-racial or Other	0.04	0.04
Income to Needs Ratio	0.66	0.71
Number of children in the home	2.59	2.71
Food Stamps	0.54	0.50
Free lunch	0.52	0.57
Bio Parent Sees Child Everyday	0.43	0.48
Married/Remarried	0.18	0.26
Parent has savings	0.66	0.57
*Child Baseline Skills*		
Executive functioning (standardized)	0.01	-0.16
Math	7.33	6.77
PPVT	10.48	9.91
*Classroom and Teacher Characteristics*		
Teacher age	37.38	43.29
Teacher depression (K6 score)	3.16	1.91
Teacher job demand	2.88	2.54
Teacher behavioral management	4.58	5.16
Classroom overall quality	4.46	4.97
F (53, 10.3) =	58.42, p < 0.001	
Number of Observations	308	294

*Note*. Descriptive characteristics for the full set of baseline covariates are presented in the supplementary information file (S3 Appendix). The F-statistic was generated by regressing treatment status on all baseline measures, and testing whether all baseline measures jointly statistically significantly differed from 0.

In general, we found that the groups were quite similar on most variables measuring demographic, parent, and home environment characteristics. However, measures of child baseline skills (e.g., executive functioning, math) tended to favor the treatment group, while measures of the preschool classroom environment (e.g., observed classroom quality) tended to favor the control group. Reflecting these differences, the F-test indicated that across all characteristics assessed at baseline, the treatment and control group significantly differed (*p <* 0.001). Thus, although the treatment program was randomly assigned, the site-paired blocking procedure was still unable to ensure perfect balance on all observable characteristics assessed. This was likely due to the relatively small number of clusters, and the high degree of variability between Head Start sites participating in the study. In the supplementary file, we present selected site-level characteristics in S4 Appendix, which further illustrates the inter-site variation (e.g., the number of children enrolled for services at the site varied from 20 to 576).

### Treatment impacts

Table 3 contains descriptive information for the outcome variables used in the treatment impact analyses, including the mean, standard deviation, and range of each variable. Scores on the Hearts and Flowers measure reflect a moderate degree of accuracy on the mixed trials task, as students correctly completed approximately 66% of trials (ranging from 6% to 100% accuracy). The mean self-reported GPA across groups was 2.8, reflecting a “C” average, and scores on the internalizing and externalizing measures both indicated a moderate degree of behavioral problems, as students indicated engagement with internalizing behaviors on approximately 30% of the items presented, and indicated engagement with externalizing behaviors on approximately 52% of the items presented.

**Table 3:** Descriptive characteristics for adolescent outcome measures.

	N	Treatment				Control			
		M	SD	Min	Max	M	SD	Min	Max
*Executive Function (H&F)*									
Mixed Trials Accuracy	460	0.67	0.19	0.06	1	0.65	0.19	0.06	1
Mixed Trials Reaction Time (Adjusted)	459	187.46	98.29	-195.29	392.67	181.83	104.92	-205.60	362.98
GPA (self-reported)	418	2.86	0.73	2.00	4.00	2.85	0.75	2.00	4.00
*Behavioral Problems*									
Internalizing	461	0.30	0.28	0	1	0.27	0.28	0	1
Externalizing	461	0.53	0.29	0	1	0.52	0.31	0	1
*Emotional Regulation (EGNG)*									
Angry D-Prime	447	1.47	0.85	-0.88	3.57	1.55	0.83	-0.94	3.57
Angry Reaction Time (Adjusted)	445	32.94	46.71	-164.13	183.16	38.29	47.44	-138.00	173.63
Sad D-Prime	447	1.35	0.83	-1.31	3.57	1.41	0.86	-1.16	3.26
Sad Reaction Time (Adjusted)	445	37.90	43.72	-73.30	222.58	39.98	42.04	-82.27	159.85

*Note*. All descriptive characteristics shown were generated from non-imputed data, and the “N” column reflects the number of non-missing cases on each measure.

Finally, the EGNG descriptive information suggests that students had some difficulty in responding accurately to Angry and Sad trials. Recall that the D-Prime scores were standardized across all blocks for each student, and a D-Prime score of “0” would indicate that students had an equal proportion of errors and correct responses when viewing emotional faces. Across the Angry and Sad trials considered here, D-Prime ranged from -1.31 to 3.57, with average performance hovering around a mean of approximately 1.4.

As Table 3 reflects, we observed few mean differences between treatment and control across the unadjusted long-term follow-up measures. However, the impact models described below show that treatment effects were detectable once adjustments for baseline differences were taken into account. Table 4 presents our treatment impact estimates on the set of long-run outcomes, and all outcomes were standardized, so coefficients can be interpreted as effect sizes. In Column 1, we present results from regression models that only included the blocking group fixed effects to adjust for the cluster-design of the study. In Column 2, we added all baseline covariates shown in S3 Appendix to adjust for differences observed between the groups at the time of random assignment. By comparing the unadjusted estimates shown in Column 1 to the estimates adjusted for baseline differences shown in Column 2, we can better understand how baseline differences between the treatment and control group might have affected the estimated treatment impacts.

**Table 4:** Impacts of the Chicago School Readiness Project on adolescent outcomes.

	No Controls	Full Controls	Full Controls/ Attrition Adjusted	Full Controls/ Site Characteristics
	(1)	(2)	(3)	(4)
*Executive Function (H&F)*				
Mixed Trials Accuracy	0.138	0.176+	0.204+	0.042
	(0.081)	(0.094)	(0.100)	(0.144)
Mixed Trials Reaction Time (adjusted)	0.072	0.010	0.001	0.053
	(0.057)	(0.078)	(0.103)	(0.134)
Self-reported GPA	0.06	0.192*	0.195	0.446**
	(0.090)	(0.087)	(0.107)	(0.139)
*Behavior Problems*				
Internalizing	0.079	-0.025	-0.033	-0.025
	(0.053)	(0.113)	(0.112)	(0.140)
Externalizing	0.028	-0.119	-0.116	0.221
	(0.098)	(0.112)	(0.131)	(0.156)
*Emotional Regulation (EGNG)*				
Angry D-Prime	-0.089	-0.159	-0.143	-0.140
	(0.079)	(0.106)	(0.106)	(0.123)
Angry RT (adjusted)	-0.094	-0.319***	-0.313*	-0.117
	(0.075)	(0.076)	(0.114)	(0.168)
Sad D-Prime	-0.028	-0.095	-0.064	-0.215
	(0.061)	(0.103)	(0.127)	(0.157)
Sad RT (adjusted)	-0.025	-0.238*	-0.231+	-0.199
	(0.029)	(0.084)	(0.109)	(0.135)
*Baseline Covariates Included*				
Blocking Group	Inc.	Inc.	Inc.	
Demographic, Family and Parent Characteristics		Inc.	Inc.	Inc.
Child Baseline Skills and Behavior		Inc.	Inc.	Inc.
Classroom/Teacher Characteristics		Inc.	Inc.	Inc.

Note. Robust standard errors were adjusted for site-level clustering in preschool and are presented in parentheses, and “Inc.” is used to denote when a particular set of control variables was included in a given regression model. All outcome variables were standardized, so coefficients can be interpreted as effect sizes. Multiple imputation (25 imputed datasets) was used to account for missing data on control variables. In Columns 1, 2, and 4, only non-missing cases on each outcome variable were considered, so sample sizes for each respective measure reflect the sample sizes listed in Table 3. In Column 3, we estimated a separate set of 25 multiply imputed datasets that included imputation on the outcome variables, so each regression model shown in Column 3 included the full sample size (n = 602).

+ p<0.10

* p< 0.05

** p < 0.01

*** p < 0.001

Beginning with H&F, our measure of executive function, we predicted that assignment to the treatment group would positively affect performance on the H&F measure, and we found some support for this hypothesis. With no covariates included, the treatment impact on H&F accuracy was positive, but not statistically significant (*ß* = 0.14, *SE* = 0.08), but this effect grew larger when covariates were added, as our fully-controlled regression model indicated a marginally statistically significant effect of 0.18 (*SE* = 0.09, *p* = 0.08). We found no indication of treatment and control differences on H&F reaction time.

For self-reported GPA, we again predicted a positive treatment effect for the CSRP group, and we again found some support for this hypothesis. With no adjustment for baseline differences, we found a treatment effect close to 0 on GPA (*ß* = 0.06, *SE* = 0.90), but this effect grew to a statistically significant effect of 0.19 (*SE* = 0.09, *p <* 0.05) once baseline differences were taken into account. Because GPA is measured on an ordinal scale, we also investigated ordinal logistic regression models to ensure that our GPA effect was not sensitive to the assumptions of linear regression. When controls were taken into account, we again found a statistically significant treatment impact (log-odds coefficient = 0.40, *SE* = 0.20, *p <* 0.05), and post-hoc marginal effects tests revealed that this impact was consistent throughout the GPA distribution.

We predicted that the treatment would lower self-report scores on our measures of externalizing and internalizing, but our models largely failed to detect differences between the treatment and control groups on these measures. When baseline differences were taken into account, we observed that adolescents in the treatment group had lower externalizing scores by about 1/10^th^ of a SD, but this effect was far from statistically significant.

Finally, we predicted that the treatment group would show a greater capacity for emotional regulation on the EGNG measure, but our findings suggested a more complex pattern of results. Estimates that included adjustments for baseline differences produced statistically significant impacts on both measures of reaction time, as treatment students had lower adjusted reaction times when viewing angry (*ß* = -0.32, *SE* = 0.08, *p <* 0.001) and sad (*ß* = -0.24, *SE* = 0.08, *p <* 0.05) faces relative to neutral faces. These results indicate that the treatment group may have exhibited heightened sensitivity to negative emotion, as students in the treatment group responded more quickly when faced with an emotionally expressive face. Interestingly, we also found negative, though not statistically significant, point estimates on both the Angry D-Prime and Sad D-Prime measures, indicating that the treatment group may have also had some difficulty with accuracy on the measure.

### Supplemental results

In the following section, we describe results from alternative statistical models that were pursued in order to confirm the reliability of the results reported in Table 4. We pursued these additional tests to examine whether our results were sensitive to statistical modelling decisions that could be viewed as arbitrary (e.g., using multiple imputation to adjust for missing data on baseline covariates instead of full information maximum likelihood) and to examine potential threats to the validity of our results due to study design shortcomings (e.g., attrition).

#### Attrition

Study attrition can potentially bias treatment impact results if attrition differentially affects the composition of the treatment or control group. Although we observed that the 23% attrition rate did not differ between the treatment and control groups (*p* = 0.51), we also investigated whether other baseline characteristics predicted attrition out of the study sample. In S6 Appendix, we present results from a linear probability regression model in which the probability of leaving the sample was modeled as a function of treatment status, blocking group, and the full set of baseline covariates. With this regression model, we again found that treatment status was not statistically significantly related to the probability of leaving the sample (*p* = 0.18), and we observed only one statistically significant predictor of attrition among all the baseline covariates investigated: children of parents who graduated from high school were more likely to leave the sample (*ß = -*0.12, *SE* = 0.05, *p <* 0.05). We also examined bivariate correlations between attrition and baseline covariates, and again found limited evidence that students who did and did not leave the sample systematically differed. These results indicated that differential attrition probably had little effect on the study results.

However, to further test the possible effect of study attrition, we replicated our main treatment impact results from Column 2 of Table 3 using 25 multiply imputed datasets that included imputation for the outcome variables of interest. In effect, these regression models use the baseline data to predict what students’ scores might have been had they remained in the sample. In Column 3 of Table 4, we present the results from these fully-controlled regression models that adjusted for attrition effects, and results were quite similar to the results shown in Column 2, again indicating a minimal impact of study attrition.

#### Site characteristics

In the original end-of-preschool treatment impact evaluations [4,5], impact estimates were derived from models that included controls for site characteristics, but did not include controls for the blocking group fixed effects. This approach has been criticized for not fully taking the clustered design of the study into account [91], which is why our preferred estimates in Column 2 include the blocking group fixed effect. However, in Column 4 of Table 4, we present estimates derived from models that included site characteristics without blocking group fixed effects to provide estimates comparable to those shown in the original evaluations. With these models, the magnitude and direction of the coefficients were largely similar to the previous specification, with two notable exceptions: the H&F mixed trials accuracy coefficient fell to a non-statistically significant 0.04 and the GPA effect more than doubled in size (*ß* = 0.47, *SE* = 0.14, *p <* 0.01). These results suggest that site level variation *within* the paired blocking groups may account for some degree of imbalance between the treatment and control groups, though the substantially larger standard errors also implies a high degree of imprecision this set of models. Unfortunately, our data were simply not adequately powered to control for a host of site characteristics without introducing substantial error.

#### Sensitivity checks

As described above, we pursued a number of sensitivity checks to ensure that arbitrary statistical decisions did not drive our results. These supplementary analyses are all presented and described in detail in the supplementary appendix (S6 Appendix), and across these models, we were looking for convergent validity to support the findings reported in Table 4. As such, we present results that used alternative methods for dealing with missing data, alternative approaches to modeling treatment impacts (i.e., Hierarchical Linear Modeling; logistic regression for GPA), and alternative measurement specifications for EGNG. Across these alternative statistical approaches, point estimates were largely similar to the estimates shown in Table 4, though standard errors did fluctuate, indicating some degree of imprecision in our results.

Finally, in the supplemental material (S7 Appendix), we also present results from various tests of treatment impact heterogeneity, and we failed to detect any consistent pattern of treatment impact heterogeneity across measures of gender, race, or poverty. We found some indication that GPA effects were largely driven by children in the first cohort, but this cohort effect was not consistently detected across other outcomes. In the supplemental material, we describe these heterogeneity tests in detail.

## Discussion

Our results provide some promising, albeit inconclusive, evidence that a high-quality, early-childhood intervention targeting classroom quality and self-regulation could produce impacts detectable during adolescence. We hypothesized that the CSRP intervention would have positive long-run effects on measures of executive function and academic achievement, and we also expected the program to reduce behavioral problems and support emotional regulation. Across our models, we found positive impacts for children who participated in the CSRP program on measures of adolescent executive functioning and academic achievement, but these effects were only detected when covariates were included. However, we found no evidence of long-run treatment effects on measures of problem behaviors. These “point in time” findings stand in contrast to earlier findings of the impact of CSRP in supporting some students to shift to more positive behavioral trajectories during elementary school [60]. This difference in result may be due to alternate analytic approaches, or due to developmental differences that occurred between elementary school and the period of adolescence considered here. In keeping with this open question, we also found that adolescents in the treatment group had heightened sensitivity to angry and sad emotional stimuli relative to the adolescents in CSRP’s control group, and these differences in emotional regulation could lead to unobserved differences in behavior.

When projecting what our results might mean for the current landscape of early interventions, it is important to recall the design of our study. Unlike other large-scale early intervention evaluations [22,26], our study did not compare students’ participation in a single ECE program versus non-participation. Rather, children assigned to the control group in our study were still enrolled in Head Start, meaning that our results inform policy conversations about models to improve Head Start rather than debates about the overall effectiveness of Head Start participation. In some respects, the fact that our study compared a Head Start improvement model versus business-as-usual Head Start constitutes a design strength, as many other ECE evaluations have struggled to accurately determine whether children in the control group sought alternative childcare arrangements or simply remained in relative care at home [16]. Thus, our study presents a clear comparison between two types of investment in the delivery of a single ECE program, but this comparison prevented us from testing whether Head Start produced long-lasting effects over alternative programs or informal early childcare arrangements.

Instead, our results suggest that efforts to improve Head Start could produce potentially beneficial long-term effects for the children already receiving Head Start services. Head Start still constitutes the largest federal investment in ECE, yet much recent attention has shifted toward developing new, state and locally funded, preschool programs [e.g., New York City, Boston, etc.]. Most recent preschool program models are more academic in focus, and they often operate inside pre-existing elementary schools, effectively adding an additional grade level prior to kindergarten. The rationale for scaling up academic preschool programs has been partly fueled by quasi-experimental evidence of the benefits of universal prekindergarten [92], correlational evidence showing the predictive importance of early academic skills [93], and because of the disappointing results of the Head Start Impact Study [94]. Moreover, policy advocates have also suggested that building new preschool programs from the ground up might be easier than trying to work within the existing structure of the Head Start system [95,17].

However, our results raise the possibility that improving Head Start, and working within the long-standing infrastructure, may be a worthwhile policy consideration. Of course, the CSRP model could not be implemented at scale without some cost, as the intervention introduced 5 professional development sessions led by trained clinicians, and treatment teachers also received extensive access to master’s level mental health consultants. While far from conclusive, our results suggest that such efforts may have led to long-term benefits for the cognitive functioning and academic achievement of low-income children facing a wide range of poverty-related stressors in their homes and neighborhoods. Certainly, future work is needed to investigate whether these results hold throughout secondary school, but these initial long-term findings imply that researchers should consider cost-effective ways to improve Head Start when engaging in discussions regarding ECE program investment.

We were somewhat surprised by our findings for the Emotional Go/ No Go (EGNG) task. On one hand, we found that children in the treatment group reacted more quickly (when adjusting for their “baseline” reaction time) in trials where they were asked to respond to angry and sad faces. On the other hand, we also found some indication that children may have had more difficulty with emotional regulation as we also observed negative D-Prime scores for children in the treatment group (though these effects were only statistically significant when using mean imputation to adjust for missing baseline data, see S6 Appendix). We did not hypothesize such a result, but taken with the reaction time findings, this implies that treated children were more alert and responsive to negative emotion as adolescents. It should be noted that a response on a direct assessment administered via a computer task is not the same as maladaptive behavior, and we found no effects of the treatment on measures of behavioral problems. When viewed alongside the impacts on executive function and academic achievement, these findings may simply indicate greater sensitivity to the presence of negative emotional stimuli. Positive changes in cognitive ability may have also led to heightened vigilance to negative emotion, which may have particular salience for CSRP students’ navigation of peer and community contexts given this sample’s relatively high exposure to violence and crime in inner-city Chicago [96].

For discussions around the long-term effects of early intervention, our findings provide an important data point, though our results were somewhat mixed. In the current study, we found null effects on measures of behavior problems, indicating fadeout, though we found positive effects on measures of executive function and achievement, indicating some degree of treatment impact persistence. It should be noted that the positive effects estimated for EF and GPA were not large, as the EF effect was approximately 1/5^th^ of a SD, and the GPA effect was similar in magnitude. For GPA, this effect size suggests a change of approximately .10 grade units, which is small, but could be important for students who face longer odds than their more economically advantaged peers when applying to, attending and persisting in college [97].

Given that the treatment considered here was a relatively limited program, and given that children in the control condition still attended Head Start, it may be surprising that we observed any differences between the treatment and control groups 10 years after the program ended. Indeed, other recent intervention evaluations using quasi-experimental designs [19,98] have also found sustained effects on measures of educational attainment and achievement, but most of these studies investigated larger programs that were compared against counterfactual conditions that included no program exposure. It is clear that we need further work to continue investigating whether the CSRP program might have sustained effects on other important dimensions of attainment, such as high school graduation and college enrollment. This work will be critical to fully understanding whether the modest gains in adolescent achievement and cognitive functioning could translate into key adult outcomes.

### Limitations

Our study is not without limitations. First, due to the developmental gap between the end-of-preschool period and the adolescent follow-up period, we could not collect the same measures in adolescence that were collected at the end of preschool. For example, end-of-preschool measures of academic achievement were simple counting and letter-naming tasks, whereas the adolescent measure of achievement was self-reported GPA. Although self-reported GPA is likely to capture academic skills that developed from the basic school-readiness skills measured in preschool, it probably also captures the degree to which adolescents have adjusted to school and their perception of their own success in their school setting. Similarly, the EF measures from the two periods were linked as they both captured attention regulation and inhibitory control, but the H&F task was measured via a computerized assessment and it also took processing speed into account. The difference was in part due to the reality that the early childhood assessment was conducted over 10 years ago, prior to the wide availability of standardized computer-based assessment tools that can be used across the lifespan such as the NIH Toolbox Dimensional Change Card Sort [99]. Despite this measurement limitation, our findings lend preliminary and promising support to the hypothesis that higher-order cognitive processes such as EF and academic achievement demonstrate continued plasticity to environmental enrichment provided early in childhood.

As with other field experiments with a relatively small number of sites to be assigned (18 Head Start centers), we found evidence that the cluster-random assignment procedure did not successfully produce treatment and control groups that were balanced across all characteristics observed at baseline. This meant that our results were highly sensitive to the inclusion of covariates, and our treatment impacts for executive function and academic achievement were only present when adjustments for baseline imbalance were included in the model. Although our fully-controlled estimates adjusted for any observable differences between treatment and control groups that could have led to bias in our long-run treatment impact estimates, we cannot rule out whether unobserved differences also biased our effects. Yet, it should be noted that our estimated effects tended to become *larger* as covariates were added, rather than smaller, introducing the distinct possibility that our findings could represent lower-bound estimates. Indeed, it is difficult to imagine a possible unobserved source of confounding variation that would have driven our estimates in the opposite direction once included, but such possibilities are merely speculative.

Further, many of our results were imprecisely estimated, and standard errors differed between models with alternative specifications. This led many of our p-values to fall within the “marginally statistically significant” range [i.e., *p <* 0.10]. While these limitations warrant concern and should be considered when interpreting our results, such issues are not unique to our study. For example, the classic evaluation studies for Perry Preschool and ABC were both plagued by attrition and small sample sizes. A recent study re-estimated the long-term treatment impacts for Perry and ABC and reported that most of the positive long-run results for both interventions were found at an alpha level of 0.10 when using a one-sided p-value test of statistical significance [16]. Thus, although our findings are merely suggestive rather than conclusive, this evidence still contributes to the existing literature base on the long-term effects of early intervention.

#### Conclusion

Our study offers preliminary and promising evidence that efforts to improve Head Start could carry important long-term positive benefits for children growing up in highly impoverished, urban communities within the U.S. Certainly, these findings warrant further investigation, and a true cost-benefit analysis of CSRP cannot be undertaken until more work uncovers whether the findings reported here extend into later periods of adolescence and adulthood. However, we believe these findings offer an important early step toward fully understanding how early intervention can affect children’s chances for long-term success.

## Supporting information

S1 Appendix Measurement information for end-of-preschool outcomes. (DOCX)

S2 Appendix Additional measurement information for self-reported GPA. (DOCX)

S3 Appendix Additional information regarding baseline equivalence. (DOCX)

S4 Appendix Site descriptive characteristics. (DOCX)

S5 Appendix Models predicting attrition. (DOCX)

S6 Appendix Analytic sensitivity checks. (DOCX)

S7 Appendix Treatment impact heterogeneity. (DOCX)
