# Supplementary material for: The Chicago School Readiness Project: Examining the long-term impacts of an early childhood intervention
Source: PLoS One. 2018 Jul 12;13(7):e0200144. doi: 10.1371/journal.pone.0200144 (PMC6042701; doi:10.1371/journal.pone.0200144)
Supplement: S1 Appendix — (DOCX) [file pone.0200144.s001.docx]

**S1 Appendix**

**Measurement Information for End-of-Preschool Outcomes**

The effects of the CSRP intervention during the Head Start year were assessed using developmentally-appropriate measures of executive function, emotional regulation, pre-academic skills, and behavior problems [1,2].

Executive function was measured with two direct assessments from the Preschool Self-Regulation Assessment (PSRA) [3]: Balance Beam [4] and Pencil Tap, adapted from the peg-tapping task [5,6] For the Balance Beam task, each child was instructed to walk a long line once, and then to walk the same line slowly. The difference between the slow and regular trials was then calculated. For the Pencil Tap task, the child was instructed to tap once when the assessor tapped twice, and tap twice when the assessor tapped once. The child’s performance on this task was assessed as the percent of correct responses. Children’s performance on these two tasks were standardized and then averaged into the “executive function” composite.

Emotional regulation, attention, and impulsivity were assessed through the 28-item PSRA Assessor Report [3]. This report was adapted from the 15-item Leiter-R social-emotional-rating subscale [7]. Additional items were added to the assessment from the Disruptive Behavior-Diagnostic Observation Schedule coding system (DB-DOS) [8]. The assessor report items were coded using a Likert scale ranging from 0 to 3, and a factor analysis yielded two factors: Attention/Impulse Control (16 items loading > .4) and Positive Emotion (7 items loading > .4).

Pre-academic skills included scores on the shortened Peabody Picture Vocabulary Test (PPVT) [9,10], a letter naming task, and the Early Math Skills assessment [11]. Each child’s proficiency in either English or Spanish was determined by their comprehension of either language in an assessor-conducted Simon Says task (PreLAS Simon Says) [12]. Children who demonstrated proficiency in English were given the PPVT, and children who were proficient in Spanish or bilingual were given the parallel Spanish-language version of the PPVT, entitled the Test de Vocabulario en Imagenes Peabody (TVIP) [13]. In both tasks, children were asked to point out one picture out of four that corresponded to the word spoken by the assessor. The letter naming assessment consisted of the letters of each alphabet (English or Spanish) divided into three groups of 8, 9, and 9 letters, arranged in approximate order of item difficulty. The Early Math Skills assessment covered basic addition and subtraction.

Behavior problems were assessed using multiple ratings from different reporters. Teachers and teaching assistants (TAs) completed the Behavior Problems Index (BPI) [14], a 28-item rating scale with items that were summed into Internalizing and Externalizing subscales. Teachers and TA’s also completed the Caregiver-Teacher Report Form (C-TRF) [15], a 100-item survey of child behaviors that was also summed into Internalizing and Externalizing subscales. For both the BPI and the C-TRF, children’s scores were averaged across the two reporters. Independent observational assessments of children’s behavior problems were conducted by CSRP research staff (blind to the treatment status of the classroom) using the Penn Interactive Peer Play Scale (PIPPS) [16,17]. The PIPPS included 30 dichotomous items for the observation of a specific behavior. These items yielded two subscales for analysis: Aggression/Disruption and Disconnection).

**References**

1. Raver CC, Jones SM, Li‐Grining C, Zhai F, Bub K, Pressler E. CSRP’s impact on low‐income preschoolers’ preacademic skills: self‐regulation as a mediating mechanism. Child Development. 2011;82(1): 362-378.
2. Raver CC, Jones SM, Li-Grining C, Zhai F, Metzger MW, Solomon B. Targeting children's behavior problems in preschool classrooms: a cluster-randomized controlled trial. Journal of Consulting and Clinical Psychology. 2009;77(2): 302.
3. Smith-Donald R, Raver CC, Hayes T, Richardson B. Preliminary construct and concurrent validity of the Preschool Self-regulation Assessment (PSRA) for field-based research. Early Childhood Research Quarterly. 2007;22(2): 173-187.
4. Murray KT, Kochanska G. Effortful control: Factor structure and relation to externalizing and internalizing behaviors. Journal of Abnormal Child Psychology. 2002;30(5): 503-514.
5. Blair C. School readiness: Integrating cognition and emotion in a neurobiological conceptualization of children's functioning at school entry. American Psychologist. 2002;57(2): 111.
6. Diamond A, Taylor C. Development of an aspect of executive control: Development of the abilities to remember what I said and to “Do as I say, not as I do”. Developmental Psychobiology. 1996;29(4): 315-334.
7. Roid GH, Miller LJ. Social emotional rating scale–Examiner version. Leiter International Performance Scale–Revised (Leiter–R) Wood Dale, IL: Stoelting; 1997.
8. Wakschlag LS, Leventhal BL, Briggs-Gowan MJ, Danis B, Keenan K, Hill C, Egger HL, Cicchetti D, Carter AS. Defining the “disruptive” in preschool behavior: What diagnostic observation can teach us. Clinical child and family psychology review. 2005;8(3): 183-201.
9. Dunn LM, Dunn LM. PPVT-III: Peabody picture vocabulary test. American Guidance Service; 1997.
10. Zill N. Letter naming task. Rockville, MD: Westat; 2003b.
11. Zill N. Early math skills test. Rockville, MD: Westat; 2003a.
12. Duncan SE, De Avila EA. PreLAS 2000. Monterey, CA: CTB/McGraw-Hill. 1998.
13. Dunn LM, Padilla ER, Lugo DE, Dunn LM. Examiner’s manual for the Test de Vocabulario en Images Peabody (Peabody Picture Vocabulary Test) Adaptacion Hispanoamericana (Hispanic American adaptation). Circle Pines, MN: American Guidance Service. 1986.
14. Zill N. Behavior problems index based on parent report. Child Trends; 1990.
15. Achenbach TM, Rescorla L. ASEBA school-age forms & profiles. 2001.
16. Fantuzzo J, Sutton-Smith B, Coolahan KC, Manz PH, Canning S, Debnam D. Assessment of preschool play interaction behaviors in young low-income children: Penn Interactive Peer Play Scale. Early Childhood Research Quarterly. 1995;10(1): 105-20.
17. Milfort R, Greenfield DB. Teacher and observer ratings of head start children’s social skills. Early Childhood Research Quarterly. 2002;17(4): 581-95.
