# Supplementary material for: The Chicago School Readiness Project: Examining the long-term impacts of an early childhood intervention
Source: PLoS One. 2018 Jul 12;13(7):e0200144. doi: 10.1371/journal.pone.0200144 (PMC6042701; doi:10.1371/journal.pone.0200144)
Supplement: S2 Appendix — (DOCX) [file pone.0200144.s002.docx]

**S2 Appendix**

**Additional Measurement Information for Self-Reported GPA**

As we detailed in the main text, we had hoped to run treatment impact models on district-reported GPA taken from administrative data. However, we only had administrative data for the students attending a school within the Chicago Public Schools (CPS) district (*n =* 314), and within this sample, CPS was only able to provide us with valid marks data for 196 students. Because an impact analysis of a treatment that was randomly assigned through clusters (i.e., blocking groups) requires a high degree of statistical power, we elected not to analyze treatment impacts on GPA taken from administrative data. Instead, we relied on self-reported GPA, and used the administrative data to validate this measurement decision.

Student-reported GPA was available for 418 adolescents, and 171 of these students also had administrative data from CPS. For students in high school, we took their overall GPA across all the courses they took during the 2015-2016 school year, and for children in middle school, we took the average of their math and reading grades. We observed a strong correlation between the average of students’ math and English grades taken from administrative data and their self-reported GPA (r(171) = 0.67, *p <* 0.001).

To test if misreporting was related to treatment status, we regressed administrative GPA on self-reported GPA, and saved the residuals from this model. We then regressed the residuals on the treatment status indicator, and found a relation of virtually 0 (*ß =* 0.04, *SE=* 0.07, *p =* 0.55), indicating that treatment status was unrelated to reporting accuracy. Finally, we found that a minority of students (*n=* 19) reported “mostly F’s” or “mostly D’s” despite having administrative records of grades closer to “C.” Further, a visual inspection of the data (see Figure S2) suggested that these 19 cases appeared as outliers when compared with the rest of the distribution. Consequently, we then recoded low grades to a “C” average, which had a marginal positive effect on the correlation between district-reported GPA and self-reported GPA (r(171) = 0.68, *p <* 0.001).

To examine if this recoding decision influenced our key treatment impact results, we also tested models that used the version of self-reported GPA that included “mostly D’s” and “mostly F’s” as the GPA outcome instead of our recoded version shown in the main text. In Table S2, we present results from models using this alternative measure of GPA (Columns 1 and 2) alongside models shown in the main text (Columns 3 and 4). As Table S2 reflects, although we found slightly smaller treatment impacts on the version of the measure that included answers for “mostly D’s” and “mostly F’s,” the treatment impact was still statistically significant at the 0.10 level and similar in magnitude to the version presented in the main text.

Figure S2

Correlation Between District-Reported GPA and Self-Reported GPA

*Note.* n=171

| Table S2 |  |  |  |  |  |
| --- | --- | --- | --- | --- | --- |
| *Impacts on Alternative Measure of Self-Reported GPA* | | | | | |
|  | GPA measure including "D" and "F" averages | | Recoded GPA measure used in main text | |  |
|  | No Controls | Full Controls | No Controls | Full Controls |  |
|  | (1) | (2) | (1) | (2) |  |
| Treatment Impact | 0.038 | 0.155+ | 0.06 | 0.192* |  |
|  | (0.083) | (0.085) | (0.090) | (0.087) |  |
|  |  |  |  |  |  |
| *Baseline Covariates Included* |  |  |  |  |  |
| Blocking Group | Inc. | Inc. | Inc. | Inc. |  |
| Demographic, Family and Parent Characteristics |  | Inc. |  | Inc. |  |
| Child Baseline Skills and Behavior |  | Inc. |  | Inc. |  |
| Classroom/Teacher Characteristics |  | Inc. |  | Inc. |  |
| *Note.* See Table 4 note. The GPA measure used in Columns 1 and 2 included responses for "mostly D's" and "mostly F's" and the GPA measure used in Columns 3 and 4 recoded the 19 cases that indicated having a "D" or "F" GPA to a "C" GPA. | | | | | |
| + p<0.10 * p< 0.05 ** p < 0.01 *** p < 0.001 | | | | |  |
