# Supplementary material for: The Chicago School Readiness Project: Examining the long-term impacts of an early childhood intervention
Source: PLoS One. 2018 Jul 12;13(7):e0200144. doi: 10.1371/journal.pone.0200144 (PMC6042701; doi:10.1371/journal.pone.0200144)
Supplement: S3 Appendix — (DOCX) [file pone.0200144.s003.docx]

**S3 Appendix**

**Additional Information Regarding Baseline Equivalence**

In Table S3, we present the full list baseline covariates used in the treatment impact models shown in Table 4. Because the list is long, we have broken the variables in to Panel A, which includes demographic and family characteristics, and Panel B, which includes baseline assessments of cognitive and behavioral skills and preschool classroom characteristics. We have also included an estimate of the blocking-group adjusted difference between the treatment and control group for each variable, and the standard error of this difference. Finally, the F-statistic presents an overall assessment of the degree to which the treatment and control group differed on the entire set of baseline covariates.

As we described in the main text, we found the set of demographic and family characteristics to be generally the same across both groups, but baseline assessments of cognitive skills tended to favor the treatment group, whereas baseline assessment of the preschool classroom tended to favor the control group. Because we found evidence of baseline differences, we control for the entire set of characteristics in our preferred treatment impact models shown in Table 4.

| Table S3 (Panel A) |  |  |  |  |
| --- | --- | --- | --- | --- |
| *Child and Parent Characteristics Measured at Baseline (PreK Entry)* | | | |  |
|  | Treatment | Control | β | SE |
| *Child Demographic Characteristics* | |  |  |  |
| Female | 0.49 | 0.58 | -0.09 | 0.01 |
| Age (years) at PreK Entry | 4.93 | 4.96 | 0.01 | 0.05 |
| African American | 0.67 | 0.64 | 0.01 | 0.04 |
| Hispanic | 0.28 | 0.27 | 0.03 | 0.07 |
| Bi-racial or Other | 0.04 | 0.04 | -0.00 | 0.02 |
| *Family/Parent Characteristics* |  |  |  |  |
| Income to Needs Ratio | 0.66 | 0.71 | -0.04 | 0.04 |
| Number of Children in the Home | 2.59 | 2.71 | -0.17 | 0.07 |
| Family Size | 4.37 | 4.46 | -0.14 | 0.08 |
| Years in Current Home | 4.11 | 4.34 | -0.34 | 0.53 |
| TANF | 0.16 | 0.13 | 0.02 | 0.03 |
| WIC | 0.28 | 0.28 | -0.02 | 0.04 |
| Food Stamps | 0.54 | 0.50 | 0.03 | 0.04 |
| Medicaid/Kidcare | 0.68 | 0.69 | -0.02 | 0.04 |
| Public Housing | 0.13 | 0.15 | -0.01 | 0.03 |
| Free/Reduced Price Lunch | 0.52 | 0.57 | -0.07 | 0.05 |
| SSI Disability | 0.09 | 0.11 | -0.02 | 0.02 |
| Family Support | 0.16 | 0.16 | 0.00 | 0.04 |
| Parent or Child is Immigrant | 0.17 | 0.22 | -0.04 | 0.05 |
| Bio Parent Sometimes Sees Child | 0.26 | 0.26 | 0.01 | 0.03 |
| Bio Parent Sees Child Everyday | 0.43 | 0.48 | -0.06 | 0.04 |
| Hours Worked per Week | 20.61 | 22.62 | -1.40 | 2.28 |
| Parent Age | 29.39 | 29.47 | -0.04 | 0.41 |
| Parent African American | 0.69 | 0.65 | 0.03 | 0.04 |
| Parent Hispanic | 0.29 | 0.29 | 0.02 | 0.06 |
| Living with Partner | 0.36 | 0.42 | -0.08 | 0.03 |
| Married/Remarried | 0.18 | 0.26 | -0.08 | 0.03 |
| Parent Has Savings | 0.66 | 0.57 | 0.10 | 0.05 |
| Parent Full-time Employed | 0.36 | 0.46 | -0.08 | 0.05 |
| Parent Unemployed | 0.39 | 0.37 | -0.00 | 0.06 |
| Mother Graduated H.S. | 0.38 | 0.39 | -0.00 | 0.03 |
| Mother Attended Some College | 0.27 | 0.29 | -0.02 | 0.04 |
| Mother Attained B.A. or Higher | 0.09 | 0.05 | 0.03 | 0.02 |
| Observations | 308 | 294 |  |  |
| *Note.* See Panel B for table note. | | | |  |

| Table 1 (Panel B) |  |  |  |  |
| --- | --- | --- | --- | --- |
| *Child Competencies and Teacher Characteristics Measured at Baseline (PreK Entry)* | | | |  |
|  | Treatment | Control | β | SE |
| *Child Baseline Skills and Behavior* |  |  |  |  |
| Executive Functioning | 0.01 | -0.16 | 0.15 | 0.07 |
| Effortful Control | -0.01 | -0.09 | 0.05 | 0.07 |
| Attention/Impulse Control | 2.25 | 2.19 | 0.05 | 0.05 |
| Positive Emotion | 2.14 | 2.12 | 0.01 | 0.05 |
| Letter Naming | 0.22 | 0.17 | 0.05 | 0.02 |
| Math | 7.33 | 6.77 | 0.55 | 0.29 |
| PPVT | 10.48 | 9.91 | 0.62 | 0.26 |
| Externalizing (Parent Report) | 7.09 | 5.79 | 1.31 | 0.39 |
| Internalizing (Parent Report) | 3.39 | 3.03 | 0.30 | 0.18 |
| Externalizing (HS Teacher Report) | 6.30 | 5.29 | 1.15 | 0.85 |
| Internalizing (HS Teacher Report) | 2.54 | 2.02 | 0.53 | 0.29 |
| *Teacher and Class Characteristics* |  |  |  |  |
| Teacher has BA | 0.73 | 0.62 | 0.06 | 0.14 |
| Teacher age | 37.38 | 43.29 | -5.65 | 2.45 |
| Techer Depression (K6 Score) | 3.16 | 1.91 | 1.45 | 0.68 |
| Teacher Job Demand | 2.88 | 2.54 | 0.37 | 0.1 |
| Teacher Job Control | 3.33 | 3.18 | 0.10 | 0.16 |
| Behavioral Management | 4.58 | 5.16 | -0.67 | 0.1 |
| Classroom Emotional Climate | 15.40 | 16.73 | -1.48 | 0.33 |
| Classroom Overall Quality | 4.46 | 4.97 | -0.47 | 0.15 |
| Class Size | 16.58 | 16.28 | 0.08 | 0.79 |
| Number of Adults in Class | 2.53 | 2.29 | 0.11 | 0.18 |
|  |  |  |  |  |
| F (53, 10.3) = | 58.42, p < 0.001 | |  | |
| Observations | 308 | 294 |  |  |
| *Note.* The values shown in the "β" and "SE" columns were derived from regressing each respective baseline variable on treatment status and a set of blocking group fixed effects. The "β" column measures the difference between the treatment and control group after adjusting for between-block differences, and the "SE" column presents the standard error of this difference. The F-statistic was generated by regressing treatment status on all baseline measures, and testing whether all baseline measures were jointly statistically significantly different from 0. | | | | |
| + p<0.10 * p< 0.05 ** p < 0.01 *** p < 0.001 | | | |  |
