# Supplementary material for: The Chicago School Readiness Project: Examining the long-term impacts of an early childhood intervention
Source: PLoS One. 2018 Jul 12;13(7):e0200144. doi: 10.1371/journal.pone.0200144 (PMC6042701; doi:10.1371/journal.pone.0200144)
Supplement: S4 Appendix — (DOCX) [file pone.0200144.s004.docx]

**S4 Appendix**

**Site Descriptive Characteristics**

In Table S4, we present descriptive characteristics (means and standard deviations) for site-level characteristics for the full sample, and then by treatment and control group status.

| Table S4 |  |  |  |
| --- | --- | --- | --- |
|  | Full Sample | Treatment | Control |
|  | M | M | M |
|  | (SD) | (SD) | (SD) |
| Number of family support workers on staff | 1.38 | 0.44 | 2.33 |
|  | (2.57) | (0.53) | (3.43) |
|  |  |  |  |
| Number of children aged 3-5 | 111.67 | 95.44 | 127.89 |
|  | (126.08) | (55.39) | (173.54) |
|  |  |  |  |
| Proportion of teachers with bachelor’s degree | 0.45 | 0.49 | 0.40 |
|  | (0.40) | (0.36) | (0.46) |
|  |  |  |  |
| Proportion of teacher assistants with college degree | 0.47 | 0.39 | 0.56 |
|  | (0.38) | (0.34) | (0.42) |
|  |  |  |  |
| Proportion of families employed | 0.71 | 0.81 | 0.62 |
|  | (0.29) | (0.22) | (0.34) |
|  |  |  |  |
| Proportion of families receiving TANF | 0.39 | 0.35 | 0.42 |
|  | (0.36) | (0.37) | (0.36) |
| Observations | 18 | 9 | 9 |

*Note.* Mean values are presented in each cell, and standard deviations are in parentheses.
