# Supplementary material for: The Chicago School Readiness Project: Examining the long-term impacts of an early childhood intervention
Source: PLoS One. 2018 Jul 12;13(7):e0200144. doi: 10.1371/journal.pone.0200144 (PMC6042701; doi:10.1371/journal.pone.0200144)
Supplement: S5 Appendix — (DOCX) [file pone.0200144.s005.docx]

**S5 Appendix**

**Site Descriptive Characteristics**

In Table S5, we present results from a linear probability model in which we modeled attrition from the sample (coded as “1” if a student had no adolescent outcome measures and “0” if they had at least one non-missing outcome) as a function of treatment status, blocking group, and all baseline covariates shown in S3 Appendix. As Table S5 reflects, only children of mother’s who graduated high school were more likely to leave the sample. No other characteristics was found to differ statistically significantly between children who left the sample and children who remained.

To ensure that the fully-controlled model did not mask important differences between students that did and did not leave the sample, we also investigated correlations between attrition and each respective baseline characteristics while controlling only for blocking group. With this set of models (not shown), we only found 3 significant predictors of attrition: 1) number of children in the home (β = -0.036, *SE =* 0.011, *p<* 0.01); 2) number of children in the home (β = -0.052, *SE =* 0.016, *p <* 0.01); 3) Preschool teacher had obtained a B.A. (β = 0.091, *SE=* 0.030, *p <* 0.01). These bivariate correlations did not indicate any substantial pattern that would suggest that students who left the sample differed systematically from students who remained.

| Table S5 |  |
| --- | --- |
| *Results from a linear probability regression model predicting attrition from the sample* | |
| Treatment | 0.075 |
|  | (0.053) |
| *Blocking Group* |  |
| 2 | -0.045 |
|  | (0.092) |
| 3 | -0.030 |
|  | (0.092) |
| 4 | -0.099 |
|  | (0.089) |
| 5 | -0.167 |
|  | (0.109) |
| 6 | 0.082 |
|  | (0.124) |
| 7 | -0.031 |
|  | (0.091) |
| 8 | -0.051 |
|  | (0.126) |
| 9 | -0.033 |
|  | (0.104) |
| *Baseline Covariates* |  |
| Female | -0.019 |
|  | (0.046) |
| Age (years) at PreK Entry | 0.022 |
|  | (0.037) |
| African American | 0.321 |
|  | (0.296) |
| Hispanic | 0.332 |
|  | (0.327) |
| Bi-racial or Other | 0.318 |
|  | (0.288) |
| Income to Needs Ratio | -0.021 |
|  | (0.022) |
| Number of Children in the Home | -0.016 |
|  | (0.023) |
| Family Size | -0.037 |
|  | (0.034) |
| Years in Current Home | -0.021 |
|  | (0.020) |
| TANF | -0.037 |
|  | (0.046) |
| WIC | 0.027 |
|  | (0.055) |
| Food Stamps | 0.067 |
|  | (0.049) |
| Medicaid/Kidcare | -0.020 |
|  | (0.056) |
| Public Housing | -0.072 |
|  | (0.058) |
| Free/Reduced Price Lunch | -0.061 |
|  | (0.053) |
| SSI Disability | -0.009 |
|  | (0.065) |
| Family Support | -0.052 |
|  | (0.049) |
| Parent or Child is Immigrant | 0.018 |
|  | (0.066) |
| Bio Parent Sometimes Sees Child | 0.008 |
|  | (0.050) |
| Bio Parent Sees Child Everyday | -0.036 |
|  | (0.058) |
| Hours Worked per Week | -0.033 |
|  | (0.069) |
| Parent Age | 0.003 |
|  | (0.017) |
| Parent African American | -0.380 |
|  | (0.271) |
| Parent Hispanic | -0.348 |
|  | (0.286) |
| Living with Partner | -0.002 |
|  | (0.065) |
| Married/Remarried | -0.005 |
|  | (0.070) |
| Parent Has Savings | 0.031 |
|  | (0.054) |
| Parent Full-time Employed | 0.060 |
|  | (0.084) |
| Parent Unemployed | 0.007 |
|  | (0.110) |
| Mother Graduated H.S. | -0.122* |
|  | (0.051) |
| Mother Attended Some College | -0.073 |
|  | (0.064) |
| Mother Attained B.A. or Higher | -0.143 |
|  | (0.087) |
| Executive Functioning | -0.008 |
|  | (0.022) |
| Effortful Control | -0.012 |
|  | (0.032) |
| Attention/Impulse Control | 0.005 |
|  | (0.027) |
| Positive Emotion | 0.027 |
|  | (0.019) |
| Letter Naming | -0.017 |
|  | (0.029) |
| Math | -0.017 |
|  | (0.035) |
| PPVT | -0.003 |
|  | (0.026) |
| Externalizing (Parent Report) | -0.017 |
|  | (0.031) |
| Internalizing (Parent Report) | 0.043 |
|  | (0.030) |
| Externalizing (HS Teacher Report) | -0.011 |
|  | (0.022) |
| Internalizing (HS Teacher Report) | -0.003 |
|  | (0.024) |
| Teacher has BA | 0.071 |
|  | (0.048) |
| Teacher age | 0.003 |
|  | (0.019) |
| Techer Depression (K6 Score) | -0.033 |
|  | (0.021) |
| Teacher Job Demand | 0.018 |
|  | (0.031) |
| Teacher Job Control | 0.011 |
|  | (0.021) |
| Behavioral Management | 0.023 |
|  | (0.044) |
| Classroom Emotional Climate | -0.019 |
|  | (0.028) |
| Classroom Overall Quality | 0.056 |
|  | (0.054) |
| Class Size | -0.015 |
|  | (0.020) |
| Number of Adults in Class | -0.027 |
|  | (0.028) |
| Constant | 0.340 |
|  | (0.175) |
| Observations | 602 |
| *Note.* Standard errors are in parentheses and were adjusted for site-level clustering. Results were generated from 25 multiply imputed datasets. | |
