# Supplementary material for: The Chicago School Readiness Project: Examining the long-term impacts of an early childhood intervention
Source: PLoS One. 2018 Jul 12;13(7):e0200144. doi: 10.1371/journal.pone.0200144 (PMC6042701; doi:10.1371/journal.pone.0200144)
Supplement: S6 Appendix — (DOCX) [file pone.0200144.s006.docx]

**S6 Appendix**

**Analytic Sensitivity Checks**

**Alternative models.** As described above, we pursued a number of sensitivity checks to ensure that arbitrary statistical decisions did not drive our results. With the results presented in Table S6.1, we were looking for convergent validity to support the findings reported in Table 4. In Columns 1 and 2, we display results from 3-level HLM models with two different baseline covariate specifications. The first model contained only blocking group, and the second model contained all baseline covariates (i.e., the same specifications used in Table 4). HLM models were run on 25 multiply imputed datasets that included imputed values for baseline covariates (but not outcome variables) using the *mi estimate: xtmixed* command in Stata 14.2. For each of the HLM models, students were entered at level 1, classrooms at level 2, and sites at level 3. Across the models, point estimates were similar to the estimates shown in Table 4, but standard errors were slightly larger in most cases. With these models, p-values for GPA and H&F accuracy were between 0.10 and 0.20, but this change in statistical significance was due to the standard error increase.

In Columns 3 and 4, we present results from structural equation models that used FIML to adjust for missing data. These models are most directly comparable to the models that adjusted for attrition in Table 4, as they included all 602 valid cases. In both respective columns, the structural equation models were run simultaneously, so each outcome variable was used as an auxiliary variable for the other outcomes. However, the standard errors in these models were not adjusted for clustering, and they were again slightly larger than the cluster-adjusted SE’s shown in Table 4. In this model, results were quite similar to the results shown in Table 4, but the GPA effect again was not statistically significant (*p =* 0.115).

Finally, in Columns 5 and 6, we used mean imputation to adjust for missing data on baseline covariates. With this method, we included a dummy variable for each variable that had missing cases, and the dummy variable was set to “1” if a student was missing a value on the corresponding baseline covariate. These models were run with the standard OLS commands in Stata 15.0, and standard errors were again adjusted for site-level clustering. Estimates were again largely similar to those shown in Table 4.

Across these models, we found a substantial degree of convergent validity for our point estimates, suggesting that the modelling approach taken in the main text did not uniquely produce our key results. However, in many cases, standard errors were larger, indicating some degree of imprecision in our estimates.

**Alternative reaction time measure.** In Table S6.2, we present models that used a recalculation of EGNG reaction times to emotional stimuli by subtracting the students’ reaction on angry or sad blocks from their respective average reaction time, and we then divided this by their standard deviation across all blocks. This recalculation aims to address potential issues with the reaction time calculation, in which “happy” trials were subtracted from the emotional stimuli reaction time, to the extent that happy trials may not represent a true baseline measure of latency. Results for recalculated reaction time suggested a similar pattern to what was presented in Table 3 of the main text, but we only found a significant treatment effect for recalculated Angry Reaction Time (β = -0.16, *SE=* 0.06, p < .05). Models for reaction time during sad trials produced negative point estimates (i.e., lower reaction times for treatment students), but these estimates were far from statistically significant. Because this measure of adjusted reaction time takes Angry, Sad, and Happy trials into account, this result really illuminates to *which* emotion students were reacting most quickly.

**Multinomial Logistic Models.** Table S6.2 displays results from multinomial logistic models for self-reported GPA. These models were run because GPA can be considered as an ordinal variable, which means that OLS models with GPA as the dependent variable violate many of the assumptions of regression (e.g., prediction out of range, heteroscedasticity, etc.). We ran logistic models using the “ologit” command in Stata 15.0, and results closely mirrored the results reported in Table 3 of the main text. For the results shown in Table S7, coefficients should be interpreted as log odds coefficients, as they measure changes in the log-odds of moving up a grade unit on the GPA variable.

| Table S6 |  | |  |  | |  | |  | |  | |  | |  |
| --- | --- | --- | --- | --- | --- | --- | --- | --- | --- | --- | --- | --- | --- | --- |
| *Impacts of the Chicago School Readiness Project on Adolescent Outcomes- Alternative Models* | | | | | | | | | | | | | | |
|  | **HLM** | | |  | | **FIML** | | | |  | | **Mean Imputation** | | |
|  | No Controls | | Full Controls |  | | No Controls | | Full Controls | |  | | No Controls | | Full Controls |
|  | (1) | | (2) |  | | (3) | | (4) | |  | | (5) | | (6) |
| *Executive Function (H&F)* |  | |  |  | |  | |  | |  | |  | |  |
| Mixed Trials Accuracy | 0.138 | | 0.176 |  | | 0.137 | | 0.214+ | |  | | 0.137 | | 0.189+ |
|  | (0.093) | | (0.121) |  | | (0.093) | | (0.117) | |  | | (0.081) | | (0.091) |
|  |  | |  |  | |  | |  | |  | |  | |  |
| Mixed Trials Reaction Time (adjusted) | 0.072 | | 0.009 |  | | 0.071 | | 0.011 | |  | | 0.071 | | 0.046 |
|  | (0.095) | | (0.128) |  | | (0.095) | | (0.123) | |  | | (0.057) | | (0.066) |
|  |  | |  |  | |  | |  | |  | |  | |  |
| Self-reported GPA | 0.060 | | 0.192 |  | | 0.060 | | 0.210 | |  | | 0.060 | | 0.163+ |
|  | (0.099) | | (0.135) |  | | (0.099) | | (0.133) | |  | | (0.090) | | (0.079) |
| *Behavior Problems* |  | |  |  | |  | |  | |  | |  | |  |
| Internalizing | 0.079 | | -0.026 |  | | 0.079 | | -0.038 | |  | | 0.079 | | 0.038 |
|  | (0.094) | | (0.119) |  | | (0.093) | | (0.117) | |  | | (0.053) | | (0.102) |
|  |  | |  |  | |  | |  | |  | |  | |  |
| Externalizing | 0.028 | | -0.121 |  | | 0.028 | | -0.111 | |  | | 0.028 | | -0.089 |
|  | (0.094) | | (0.126) |  | | (0.094) | | (0.124) | |  | | (0.098) | | (0.103) |
|  |  | |  |  | |  | |  | |  | |  | |  |
| *Emotional Regulation (EGNG)* |  | |  |  | |  | |  | |  | |  | |  |
| Angry D-Prime | -0.089 | | -0.160 |  | | -0.089 | | -0.140 | |  | | -0.089 | | -0.189+ |
|  | (0.096) | | (0.127) |  | | (0.096) | | (0.124) | |  | | (0.079) | | (0.101) |
|  |  | |  |  | |  | |  | |  | |  | |  |
| Angry RT (adjusted) | -0.094 | | -0.319* |  | | -0.093 | | -0.334** | |  | | -0.093 | | -0.324*** |
|  | (0.103) | | (0.131) |  | | (0.097) | | (0.130) | |  | | (0.075) | | (0.075) |
|  |  | |  |  | |  | |  | |  | |  | |  |
| Sad D-Prime | -0.028 | | -0.096 |  | | -0.028 | | -0.078 | |  | | -0.028 | | -0.067 |
|  | (0.096) | | (0.127) |  | | (0.096) | | (0.124) | |  | | (0.061) | | (0.104) |
|  |  | |  |  | |  | |  | |  | |  | |  |
| Sad RT (adjusted) | -0.025 | | -0.236+ |  | | -0.025 | | -0.247+ | |  | | -0.025 | | -0.221* |
|  | (0.097) | | (0.130) |  | | (0.097) | | (0.129) | |  | | (0.029) | | (0.077) |
|  |  | |  |  | |  | |  | |  | |  | |  |
| *Baseline Covariates Included* |  | |  |  | |  | |  | |  | |  | |  |
| Blocking Group | Inc. | | Inc. |  | | Inc. | | Inc. | |  | | Inc. | | Inc. |
| Dem., Family and Parent Characteristics |  | | Inc. |  | |  | | Inc. | |  | |  | | Inc. |
| Child Baseline Skills and Behavior |  | | Inc. |  | |  | | Inc. | |  | |  | | Inc. |
| Classroom/Teacher Characteristics |  | | Inc. |  | |  | | Inc. | |  | |  | | Inc. |
| *Note.* All outcome variables were standardized, so coefficients can be interpreted as effect sizes. For estimates shown in Columns 1 and 2, multiple imputation (25 imputed datasets) was used to account for missing data on control variables, and only non-missing cases on each outcome variable were considered (sample sizes for each respective measure reflect the sample sizes listed in Table 3). In Columns 3 and 4, we estimated structural equation models using the FIML estimator in Stata 15.0. For each estimate shown, the other outcome measures were used as auxiliary variables, and all cases were considered (*n* = 602). For the estimates shown in Columns 5 and 6, missing cases on control variables were imputed using the mean value for each variable, and "missing dummy variables" (explained in S6 Appendix Text) were used to adjust for this imputation procedure. | | | | | | | | | | | | | | |
| Table S6.2 | |  | | |  | |  | |  | |  | |  |  |
| *Treatment Impacts for Alternative Specifications of EGNG Reaction Time* | | | | | | |  | |  | |  | |  |  |
|  | | Angry Reaction Time (Adjusted) | | | | |  | | Sad Reaction Time (Adjusted) | | | |  |  |
|  | | No Controls | | | Full Controls | |  | | No Controls | | Full Controls | |  |  |
|  | | (1) | | | (2) | |  | | (3) | | (4) | |  |  |
| Treatment Impacts | | -0.014 | | | -0.157* | |  | | -0.001 | | -0.046 | |  |  |
|  | | (0.052) | | | (0.063) | |  | | (0.056) | | (0.071) | |  |  |
|  | |  | | |  | |  | |  | |  | |  |  |
| *Baseline Covariates Included* | |  | | |  | |  | |  | |  | |  |  |
| Blocking Group | | Inc. | | | Inc. | |  | | Inc. | | Inc. | |  |  |
| Demographic, Family and Parent Characteristics | |  | | | Inc. | |  | |  | | Inc. | |  |  |
| Child Baseline Skills and Behavior | |  | | | Inc. | |  | |  | | Inc. | |  |  |
| Classroom/Teacher Characteristics | |  | | | Inc. | |  | |  | | Inc. | |  |  |
| *Note.*  n=447. Standard errors are in parentheses. Reaction time was calculated by taking each student’s reaction time on either angry or sad blocks and subtracting it from their respective average reaction time across all blocks. This difference score was then divided by their standard deviation across all blocks to create an adjusted reaction time value. Estimates were derived using the "mean imputation" method shown in Table S6.2. | | | | | | | | | | | | |  |  |
| + p<0.10 * p< 0.05 ** p < 0.01 *** p < 0.001 | | | | | | |  | |  | |  | |  |  |

| Table S6.3 |  |  |
| --- | --- | --- |
| *Multinomial Logistic Regression Analyses: Treatment Impacts of Self-Reported GPA* | | |
|  | GPA | |
|  | No Controls | Full Controls |
|  | (1) | (2) |
| Treatment Impacts | 0.125 | 0.403* |
|  | (0.171) | (0.201) |
|  |  |  |
| *Baseline Covariates Included* |  |  |
| Blocking Group | Inc. | Inc. |
| Demographic, Family and Parent Characteristics |  | Inc. |
| Child Baseline Skills and Behavior |  | Inc. |
| Classroom/Teacher Characteristics |  | Inc. |
| *Note.*  n=447. Standard errors are in parentheses. Reaction time was calculated by taking each student’s reaction time on either angry or sad blocks and subtracting it from their respective average reaction time across all blocks. This difference score was then divided by their standard deviation across all blocks to create an adjusted reaction time value. Estimates were derived using the "mean imputation" method shown in Table S6.1. | | |
| + p<0.10 * p< 0.05 ** p < 0.01 *** p < 0.001 | | |
