# Supplementary material for: The Chicago School Readiness Project: Examining the long-term impacts of an early childhood intervention
Source: PLoS One. 2018 Jul 12;13(7):e0200144. doi: 10.1371/journal.pone.0200144 (PMC6042701; doi:10.1371/journal.pone.0200144)
Supplement: S7 Appendix — (DOCX) [file pone.0200144.s007.docx]

**S7 Appendix**

**Treatment Impact Heterogeneity**

In the supplementary Table S7.1, we present results from models that tested for treatment impact heterogeneity based on gender, race, and exposure to extreme poverty during the Head Start year, which was defined as having an unemployed mother that did not graduate high school and having family income no higher than 50% of the poverty line. We pursued these heterogeneity tests as they were in keeping with the set of moderators tested in the original treatment impact evaluation for end-of-preschool outcomes [8].

All models shown in Table S7 included blocking group, demographic and parent characteristics, baseline skills and behavior, and classroom/teacher characteristics. The moderating effects of student gender, race, and poverty on treatment, as well as the main effects are presented in the table. Examining the coefficients across the rows, although there are a few significant moderating effects of race and poverty, there is no evidence of consistent heterogeneity by these variables.

Table S7.2 presents heterogeneity by cohort status, presented as 9 fully-controlled regression models with site-clustered standard errors. The *Treatment*Cohort* variable displays significant evidence of moderation for the outcome of GPA (β = 0.65, p < .001), suggesting that the treatment impact of GPA was driven by students in the first cohort. We also observed a marginally statistically significant interaction for cohort status for EGNG angry reaction time (β = -0.35, p < .10), but this same effect was not observed for reaction time on sad trials, making it difficult to draw a strong conclusion regarding impact heterogeneity on EGNG performance.

The cohort effect could also be due to differences in grade level at the time of follow-up assessment. Indeed, 90% of children in the first cohort were in high school at the time of assessment, whereas the majority of children in the second cohort were in middle school. However, because cohort status was so highly correlated with adolescent grade level, only collecting data from future waves will allow us to test if this effect was driven by students attending high school rather than cohort status itself.

| Table S7.1 |  |  |  |  |  |  |  |  |  |
| --- | --- | --- | --- | --- | --- | --- | --- | --- | --- |
| *Heterogeneity Test: Treatment Interactions with Key Demographic Variables* | | | | | | | | | |
|  | Hearts and Flowers | | Grades | Behavior Problems | | Emotional Regulation (EGNG) | | | |
|  | Accuracy | Reaction Time | Self-reported GPA | Internalizing | Externalizing | Angry D-Prime | Angry RT | Sad D-Prime | Sad RT |
| Treatment | 0.372* | 0.127 | 0.415* | 0.134 | 0.188 | 0.218 | -0.508+ | 0.231 | 0.043 |
|  | (0.164) | (0.189) | (0.189) | (0.204) | (0.228) | (0.160) | (0.246) | (0.195) | (0.162) |
| *Interactions* |  |  |  |  |  |  |  |  |  |
| Treatment * Female | 0.194 | 0.218 | -0.209 | 0.078 | -0.232 | -0.291 | 0.149 | -0.333+ | 0.010 |
|  | (0.142) | (0.163) | (0.201) | (0.228) | (0.153) | (0.186) | (0.181) | (0.183) | (0.147) |
| Treatment * Black | -0.379 | -0.270 | -0.182 | -0.234 | -0.194 | -0.362+ | 0.164 | -0.218 | -0.373+ |
|  | (0.226) | (0.213) | (0.233) | (0.218) | (0.242) | (0.177) | (0.286) | (0.287) | (0.214) |
| Treatment * Poverty | -0.486 | -0.267 | -0.494 | 0.452 | -0.412 | -0.082 | -0.140 | 0.592+ | -0.313 |
|  | (0.280) | (0.529) | (0.309) | (0.467) | (0.429) | (0.323) | (0.370) | (0.339) | (0.395) |
| *Main Effects* |  |  |  |  |  |  |  |  |  |
| Female | -0.268** | -0.091 | 0.273 | 0.643** | 0.208* | 0.417** | -0.045 | 0.124 | 0.140 |
|  | (0.089) | (0.112) | (0.163) | (0.197) | (0.091) | (0.124) | (0.107) | (0.152) | (0.108) |
| Black | 0.257 | 0.565 | 0.443 | -0.328 | 0.060 | 0.014 | 0.116 | 0.078 | -0.003 |
|  | (0.164) | (0.375) | (0.267) | (0.314) | (0.364) | (0.215) | (0.264) | (0.286) | (0.367) |
| Poverty | -0.871 | -0.761 | 0.228 | -0.326 | -0.559 | 1.341+ | -0.312 | 1.103 | 0.714 |
|  | (0.727) | (0.662) | (0.581) | (0.540) | (0.618) | (0.730) | (0.477) | (1.171) | (0.664) |
| *Baseline Covariates Included* |  |  |  |  |  |  |  |  |  |
| Blocking Group | Inc. | Inc. | Inc. | Inc. | Inc. | Inc. | Inc. | Inc. | Inc. |
| Demographic & Family Characteristics | Inc. | Inc. | Inc. | Inc. | Inc. | Inc. | Inc. | Inc. | Inc. |
| Child Baseline Skills & Behavior | Inc. | Inc. | Inc. | Inc. | Inc. | Inc. | Inc. | Inc. | Inc. |
| Classroom/Teacher Characteristics | Inc. | Inc. | Inc. | Inc. | Inc. | Inc. | Inc. | Inc. | Inc. |
| Observations | 460 | 459 | 418 | 461 | 461 | 447 | 445 | 447 | 445 |
| *Note.* Standard errors are shown in parentheses and were adjusted for site-level clustering. The "poverty" variable in the interaction term is an aggregate of cumulative exposure to three poverty-related risks, including mother's educational attainment of less than a high school degree, family income-to-needs ratio for the previous year being less than half the federal poverty threshold, and mother's engagement in 10 hours or fewer of employment per week. Estimates were derived using the "mean imputation" method shown in Table S6.1. | | | | | | | | | |
| + p<0.10 * p<0.05 ** p<0.01 *** p<0.001 | | | | | | | | | |

| Table S7.2 |  |  |  |  |  |  |  |  |  |
| --- | --- | --- | --- | --- | --- | --- | --- | --- | --- |
| *Heterogeneity Test: Treatment Interactions with Cohort Status* | | | | | | | | | |
|  | Hearts and Flowers | | Grades | Behavior Problems | | Emotional Regulation (EGNG) | | | |
|  | Accuracy | Reaction Time | Self-reported GPA | Internalizing | Externalizing | Angry D-Prime | Angry RT | Sad D-Prime | Sad RT |
| Treatment | 0.144 | -0.116 | -0.219 | 0.058 | -0.129 | -0.404* | -0.115 | 0.125 | -0.148 |
|  | (0.152) | (0.134) | (0.153) | (0.143) | (0.118) | (0.156) | (0.119) | (0.177) | (0.114) |
|  |  |  |  |  |  |  |  |  |  |
| Treatment * Cohort | 0.078 | 0.279 | 0.651*** | -0.034 | 0.068 | 0.365 | -0.353+ | -0.326 | -0.124 |
|  | (0.178) | (0.199) | (0.154) | (0.153) | (0.205) | (0.232) | (0.173) | (0.238) | (0.138) |
| *Baseline Covariates Included* |  |  |  |  |  |  |  |  |  |
| Blocking Group | Inc. | Inc. | Inc. | Inc. | Inc. | Inc. | Inc. | Inc. | Inc. |
| Demographic & Family Characteristics | Inc. | Inc. | Inc. | Inc. | Inc. | Inc. | Inc. | Inc. | Inc. |
| Child Baseline Skills & Behavior | Inc. | Inc. | Inc. | Inc. | Inc. | Inc. | Inc. | Inc. | Inc. |
| Classroom/Teacher Characteristics | Inc. | Inc. | Inc. | Inc. | Inc. | Inc. | Inc. | Inc. | Inc. |
|  |  |  |  |  |  |  |  |  |  |
| Observations | 460 | 459 | 418 | 461 | 461 | 447 | 445 | 447 | 445 |
| *Note.* Standard errors are shown in parentheses and were adjusted for site-level clustering. As with our other key models, cohort was not entered as a covariate because it is captured by the blocking group fixed effects. These models were run using the “mean imputation” method shown in Table S6.1. | | | | | | | | | |
| + p<0.10 * p<0.05 ** p<0.01 *** p<0.001 | | | | | | | | | |
